# Macrophage Targeting Protects Nerve Structure and Improves Muscle Innervation in a Mouse Model of Charcot‐Marie‐Tooth 2J

**DOI:** 10.1002/glia.70074

**Published:** 2025-08-04

**Authors:** Dennis Klein, Neslim Ercan, Xidi Yuan, Ghjuvan' Ghjacumu Shackleford, Anke Claessens, M. Laura Feltri, Lawrence Wrabetz, Maurizio D'Antonio, Rudolf Martini

**Affiliations:** ^1^ Department of Neurology, Developmental Neurobiology University Hospital Würzburg Würzburg Germany; ^2^ Biology of Myelin Unit, Division of Genetics and Cell Biology San Raffaele Scientific Institute Milan Italy; ^3^ Department of Neurology, Institute for Myelin and Glia Exploration, Jacobs School of Medicine and Biomedical Sciences State University of New York at Buffalo Buffalo New York USA; ^4^ Department of Biochemistry, Institute for Myelin and Glia Exploration, Department Biochemistry and Neurology, Jacobs School of Medicine and Biomedical Sciences State University of New York at Buffalo Buffalo New York USA

**Keywords:** axon degeneration, axonopathy, colony stimulating factor 1, inherited peripheral neuropathy, macrophage, neuroinflammation

## Abstract

In several previous studies, we have shown that macrophage targeting with the CSF‐1 receptor specific kinase (c‐FMS) inhibitor PLX5622 led to a substantial alleviation of the neuropathy in distinct mouse models of demyelinating Charcot‐Marie‐Tooth (CMT) 1 forms. However, whether macrophages are also relevant drivers of the neuropathy in axonal CMT2 subtypes has not been studied so far. Here, we investigated the role of macrophages in hemizygous P0T124M mice, which develop a late‐onset axonopathy accompanied by macrophage activation at 18 months of age and reflect typical pathological signs of a CMT2J neuropathy. As a tool to target macrophages before disease onset, hemizygous P0T124M mice were treated with PLX5622 from 12 to 18 months of age. Remarkably, treatment with PLX5622 not only ameliorated the peripheral neuropathy to an exceptionally high degree but also prevented distal axonal degeneration and denervation of neuromuscular junctions, leading to preserved motor function in CMT2J mice. These findings highlight macrophage‐mediated inflammation as a treatment target in peripheral nerves not only in previously investigated demyelinating but also in axonal CMT neuropathies.

## Main Points

- Hemizygous POT124M mice develop a late‐onset axonopathy at 18 months of age.
- Pharmacological macrophage targeting with PLX5622 prevents distal axon degeneration in POT124M mutant mice and mitigates motor impairment.

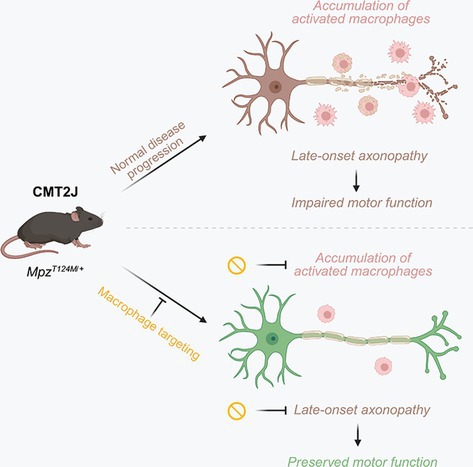

## 1 Introduction

Charcot‐Marie‐Tooth (CMT) diseases comprise a large group of inherited peripheral neuropathies, primarily affecting either Schwann cells (demyelinating CMT1) or axons (axonal CMT2) (Jerath and Shy 2015; Pisciotta and Pareyson 2023; Pisciotta et al. 2021a, 2021b). While immune cells, particularly endoneurial macrophages, are well known to contribute to the pathogenesis of several demyelinating forms (Bolino and D'Antonio 2023; Klein and Martini 2016; Martini et al. 2013; Martini and Willison 2016) their role in axonal CMT remains less understood (Kaminska and Kochanski 2024). Recent studies in models for de‐ and dysmyelinating CMT showed that macrophages are likely involved in secondary axonal damage (Berghoff et al. 2005; Ey et al. 2007; Groh et al. 2010; Groh et al. 2012), but whether they play a pathogenic role in CMT2, which is characterized by axonopathy while myelin sheets are largely preserved, remains an open question.

Mutations in the *MPZ* gene generally cause the demyelinating subtype CMT1B (Callegari et al. 2019; Sanmaneechai et al. 2015). However, certain *MPZ* mutations have been linked to axonal forms of CMT, such as the missense mutation that replaces threonine with methionine at position 124 (P0T124M), leading to late‐onset CMT2J disease (Chapon et al. 1999; De Jonghe et al. 1999; Hanemann et al. 2001; Kurihara et al. 2003). Recently, a mouse model was developed to study neuropathic changes and characterize axo‐glial communication in P0T124M mutant mice (Shackleford et al. 2022). Indeed, several pathological features and clinical aspects that are also observed in CMT2J patients could be described in the P0T124M mutant mice. However, these investigations primarily focused on homozygous P0T124M mutants (TM/TM) (Shackleford et al. 2022) displaying a more severe and early‐onset phenotype.

Since most CMT2J patients carry only one mutated allele (Kurihara et al. 2003; Misu et al. 2000), we conducted a detailed study on hemizygous P0T124M mutant mice to further explore axonopathic alterations during disease progression and to investigate the functional role of macrophage activation in axonal CMT.

Here we demonstrate that hemizygous P0T124M mutant mice (*Mpz* ^T124M/+^, TM/+) develop a late‐onset axonopathy, specifically mimicking the neuropathy observed in CMT2J patients (De Jonghe et al. 1999; Hanemann et al. 2001). At 18 months of age, TM/+ mice exhibit progressive axonal degeneration, with typical pathological hallmarks identified at both the light‐ and electron‐microscopic levels. Importantly, this was accompanied by an increased number of macrophages displaying features of activation, suggesting that macrophages might act as a potential driver of disease progression. To test this hypothesis, we targeted macrophages by treating TM/+ mice with the CSF‐1R inhibitor PLX5622 from 12 to 18 months of age. Supporting our hypothesis of macrophage‐mediated axon damage in our CMT2J model, macrophage targeting prevented distal axonal degeneration and preserved motor performance and nerve function.

Our studies demonstrate for the first time that macrophages actively contribute to the pathogenesis of axonal CMT and, consequently, support macrophage targeting as a potential treatment option for mitigating peripheral neuropathy.

## 2 Material and Methods

### 2.1 Mice

Hemizygous P0T124M (= Mpz^T124M/+^ = TM/+) (Shackleford et al. 2022), WT and PLX5622‐treated mice of both sexes were investigated at 18 months of age. All mice were on a C57Bl6/J background. Genotyping was done by conventional PCR with the use of isolated DNA from mice ear biopsies based on previously published protocols (Shackleford et al. 2022). Animals were kept in individually ventilated cages (IVC) under barrier conditions at the Center of Experimental Molecular Medicine, University of Würzburg, with a 14/10 h day/night rhythm (< 300 lux during day). Colonies were maintained at 20°C–24°C and 40%–60% humidity, with free access to food and water. All animal experiments were approved by the local authority of the Government of Lower Franconia, Germany.

### 2.2 PLX5622 (CSF‐1 Receptor Inhibitor) Treatment

Rodent chow containing 300 mg/kg CSF‐1 receptor specific kinase (c‐FMS) inhibitor (termed hereafter PLX5622) was provided by Plexxikon Inc. Mice were separated into two groups in terms of receiving the PLX5622 treatment. P0T124M mice were treated with PLX5622 from 12 to 18 months of age. Hemizygous P0T124M control mice received normal chow during the duration of the whole project (Figure S2A). During experiments, animals were monitored individually. No mice had to be excluded based on problems in health and behavior.

### 2.3 Nerve Dissection and Tissue Processing

Animals were sacrificed by asphyxiation (CO_2_) in accordance with guidelines by the State Office of Health and Social Affairs, Berlin, as previously published (Klein et al. 2022; Klein et al. 2024). Mice were then perfused transcardially using phosphate‐buffered saline (PBS) containing heparin. To harvest fresh frozen tissue for immunohistochemistry, femoral nerves (comprising the quadriceps and saphenous branch), spinal roots (L3–L5) and flexor digitorum brevis (FDB) muscles of P0T124M mutants and WT mice were embedded in Tissue‐Tek O.C.T. compound (Sakura) and frozen in liquid nitrogen‐cooled methyl butane. Fresh frozen tissues were then cut into 10‐μm‐thick cross‐sections using a cryostat (Leica) and stored at −20°C until further analysis.

For electron microscopy, mice were transcardially perfused with 4% PFA and 2% glutaraldehyde in 0.1 M cacodylate buffer for 10 min. Dissected femoral nerves and spinal roots (L3—L5) were subsequently post‐fixed in the same solution overnight at 4°C. Osmification was performed with 2% osmium tetroxide in 0.1 M cacodylate buffer for 2 h, followed by dehydration in ascending acetone concentrations. Nerves were embedded in Spurr's medium. Ultrathin sections (70 nm) were cut and mounted to copper grids and counterstained with lead citrate.

### 2.4 Immunohistochemistry

Quantification of total numbers and the assessment of activation of endoneurial macrophages were performed on cross sections of fresh frozen femoral nerves of mice.

To quantify homeostatic macrophages, samples were blocked with 5% bovine serum albumin (BSA) in PBS and incubated at 4°C with a primary antibody against CD163 (rat, 1:250, 14‐1631‐82, Life Technologies) in 1% BSA in PBS overnight. After washing with PBS, samples were incubated with AF488‐conjugated goat anti‐rat IgG (1:300, A11006, Invitrogen) secondary antibodies in 1% BSA in PBS for 1 h at room temperature. After an avidin‐biotin blocking step (Vector Laboratories) biotinylated rat anti‐mouse F4/80 (1:200, MCA497B, Serotec) primary antibodies were incubated in 1% BSA in PBS at 4°C overnight and visualized using Cy3‐conjugated Streptavidin (1:100, CLCSA1010, Cedarlane) in 1% BSA in PBS for 1 h at room temperature. To additionally assess homeostatic macrophages, the same staining protocol was applied using a primary antibody against Mgl1 (Clec10a, rat, 1:200, 145,602, Biolegend) with the following modifications: samples were additionally postfixed in acetone (10 min, −20°C) and blocked with 5% BSA with 0.1% Triton X‐100 in PBS for 1 h at room temperature.

To quantify activated macrophages in femoral nerves, samples were postfixed in acetone (10 min, −20°C), blocked with 5% BSA with 0.3% Triton X‐100 in PBS for 1 h at room temperature, and incubated with antibodies against F4/80 (rat, 1:300, MCAP497, Serotec) and Gpnmb (rabbit; 1:300, ab188222, Abcam) in 1% BSA in PBS overnight at 4°C. After washing with PBS, samples were incubated with Cy3‐conjugated donkey anti‐rat IgG (1:300, 712‐165‐163, Dianova) and AF488‐conjugated donkey anti‐rabbit IgG (1:300, 711‐545‐152, Dianova) secondary antibodies for 1 h at room temperature.

Nuclei were stained with DAPI (Sigma‐Aldrich) and slides were mounted with Aqua‐Poly/Mount (Polysciences). The mean numbers of F4/80‐positive profiles and CD163+/F480+, Mgl1+/F4/80+ or Gpnmb+/F4/80+ double‐positive macrophages, respectively, in both quadriceps and saphenous nerves were determined in seven to ten sections per animal. Additionally, the percentages of homeostatic (CD163+/F480+ and Mgl1+2/F4/80+) and activated (Gpnmb+/F4/80+) macrophages were calculated accordingly.

Muscle innervation was determined as previously reported (Klein et al. 2024; Ostertag et al. 2022). After blocking with 5% BSA with 0.3% Triton X‐100 in PBS, cross sections of the FDB muscle were incubated with a primary antibody against synaptophysin (guinea pig, 1:500, 101,004, Synaptic Systems) in 1% BSA with 0.3% Triton X‐100 at 4°C overnight, followed by visualization of presynaptic terminals using Cy3‐conjugated donkey anti‐guinea pig IgG secondary antibody (1:300, 706‐165‐148, Dianova). Postsynapses were labeled with AlexaFluor488‐conjugated α‐bungarotoxin (1:300, B‐13422, Invitrogen). At least 100 neuromuscular junctions (NMJs) were analyzed per animal. Innervated NMJs were identified by the complete overlap of synaptophysin with α‐bungarotoxin labeling (Klein et al. 2024; Ostertag et al. 2022). Partially denervated NMJs were identified by an overlay of postsynaptic labeling with incomplete presynaptic staining. Denervated NMJs were identified by the absence of presynaptic staining. The latter two were summarized as “abnormally innervated NMJs” (Klein et al. 2024; Ostertag et al. 2022).

Digital fluorescence microscopic images were acquired using an Axiophot 2 microscope (Zeiss) equipped with a CCD camera (Visitron Systems) or an Axio Imager M2 microscope (Zeiss) with ApoTome.2 structured illumination equipment, attached Axiocam cameras, and corresponding software (ZEN v.2.3 blue edition). Images were minimally processed for the generation of figures using Photoshop (Adobe) or Fiji/Image J (National Institutes of Health).

### 2.5 Morphological Analysis by Electron Microscopy

Electron microscopic images were taken using a ProScan Slow Scan CCD camera mounted to a Leo 906E electron microscope (Carl Zeiss). Multiple images were aligned with the software iTEM (Olympus Soft Imaging Solutions) to analyze whole nerve sections. Pathological alterations were determined in relation to the total number of axons in corresponding cross sections of the femoral quadriceps nerve and lumbar ventral roots. Axons with abnormal myelin (axons devoid of myelin and thinly myelinated axons), regeneration clusters, degenerating or degenerated axons, Büngner cells, myelin vacuoles, and periaxonal vacuoles were determined and then quantified. Degenerating axons were defined as profiles with cytoskeletal disorganization (i.e., microtubule and neurofilament breakdown), dark appearance, and disintegration or fragmentation in degenerating axons associated with an intact or nearly intact myelin sheath (see Figure 2). Additionally, phagocytosing macrophages were counted, and their numbers were given as relative values per 100 axons within the nerve. In the case of ventral roots, semithin sections were analyzed. Representative micrographs of indicated pathological features have been previously published (Groh et al. 2016; Klein et al. 2024; Yuan et al. 2018).

### 2.6 Nerve Conduction Studies

Mice were anesthetized by an intraperitoneal injection of a mixture of Ketavet (Pfizer) and Xylavet (CP‐Pharma) (100 mg Ketavet and 6.7 mg Xylavet per kilogram bodyweight) and were placed under a heating lamp to avoid hypothermia. Body temperature of mice was monitored before and after measurements (34°C–36°C). Electrophysiological properties of the left sciatic nerve were measured as described before (Klein et al. 2021; Zielasek et al. 1996). To measure compound muscle action potentials (CMAPs) and nerve conduction velocity (NCV), hind paw muscles of mice were recorded using steel needle electrodes. Distal and proximal latencies as well as the distance between stimulation sites were measured. The corresponding NCV was calculated by the distance between stimulation sites divided by the difference between proximal and distal latencies. Moreover, F wave latencies were recorded. All neurographic recordings were performed via a digital Neurosoft‐Evidence 3102 electromyograph (Schreiber & Tholen Medizintechnik) and the mean of five recordings per animal was measured for subsequent analysis.

### 2.7 Motor Performance Tests (Grip Strength, Rotarod and Beam Walk)

Grip strength of hindlimbs was measured using an automated Grip Strength Meter (Columbus Instruments) as described previously (Ostertag et al. 2022). With forelimbs supported by a metal grid, mice were trained to hold a grip bar properly with hind paws. Mice were pulled off the grip bar with constant strength to measure the maximum force (in newton). Ten measurements per day were performed on three consecutive days, and the mean of all measurements was calculated.

For rotarod analysis, mice were placed on a rod from a RotaRod Advanced System (TSE Systems) as described previously (Klein et al. 2021) and were trained to walk on the accelerating rod (5–50 rpm; maximum latency: 300 s) until falling off. Five training runs were performed on two consecutive days, and the latency to fall was measured for five runs on the third day.

Beam walk analysis was performed as previously reported (Klein et al. 2024). During two consecutive days of training, mice had to cross a 30‐mm‐wide beam over 90 cm to reach a platform. On the testing day (Day 3), each mouse performed the test similarly but crossed a 10‐mm‐wide beam instead. Three valid runs (constant speed along the 70 cm long recording distance) per animal were recorded, and the latency to cross the beam and the number of foot slips were determined.

### 2.8 Statistical Analyses

All experiments were performed with the investigators blinded and not aware of the genotype and experimental status of the analyzed animals. Animals were randomly placed into experimental or control groups according to genotyping results using a random generator (http://www.randomizer.org). Biometrical sample size estimation was performed with G*Power (Faul et al. 2007). Calculation of sample appropriate size groups was performed using a priori power analysis with a defined adequate power of 0.8 (1—beta error) and an α error of 0.05. To determine the prespecified effect size *d*, previously published data were considered as comparable reference values (Klein et al. 2024; Klein et al. 2021). Data sets were tested for normal distribution and variance homology. Normally distributed data was further analyzed by one‐way ANOVA and Tukey's multiple comparison test; not normally distributed data was analyzed by Kruskal‐Wallis and Dunn's multiple comparisons test. Significance levels (* *p* < 0.05, ** *p* < 0.01, *** *p* < 0.001) and statistical tests are indicated together in the figure legends. For analyzing longitudinal measurements of 12‐, 15‐, and 18‐month‐old mice, two‐way ANOVA and Tukey's multiple comparison tests were performed. Significant differences to 12‐month‐old genotype‐matched samples (WT or TM/+) are indicated by red asterisks, and significant differences between age‐matched samples (WT vs. TM/+) are indicated by black asterisks. Measurements and quantifications are shown as individual values (n‐numbers are represented in the graphs as circles) and mean ± SD, unless stated otherwise. All statistical analyses and generation of graphs were performed with GraphPad Prism (Version 8).

## 3 Results

### 3.1 Hemizygous P0T124M (TM/+) Mice Develop a Late‐Onset Axonopathy

**FIGURE 1:**
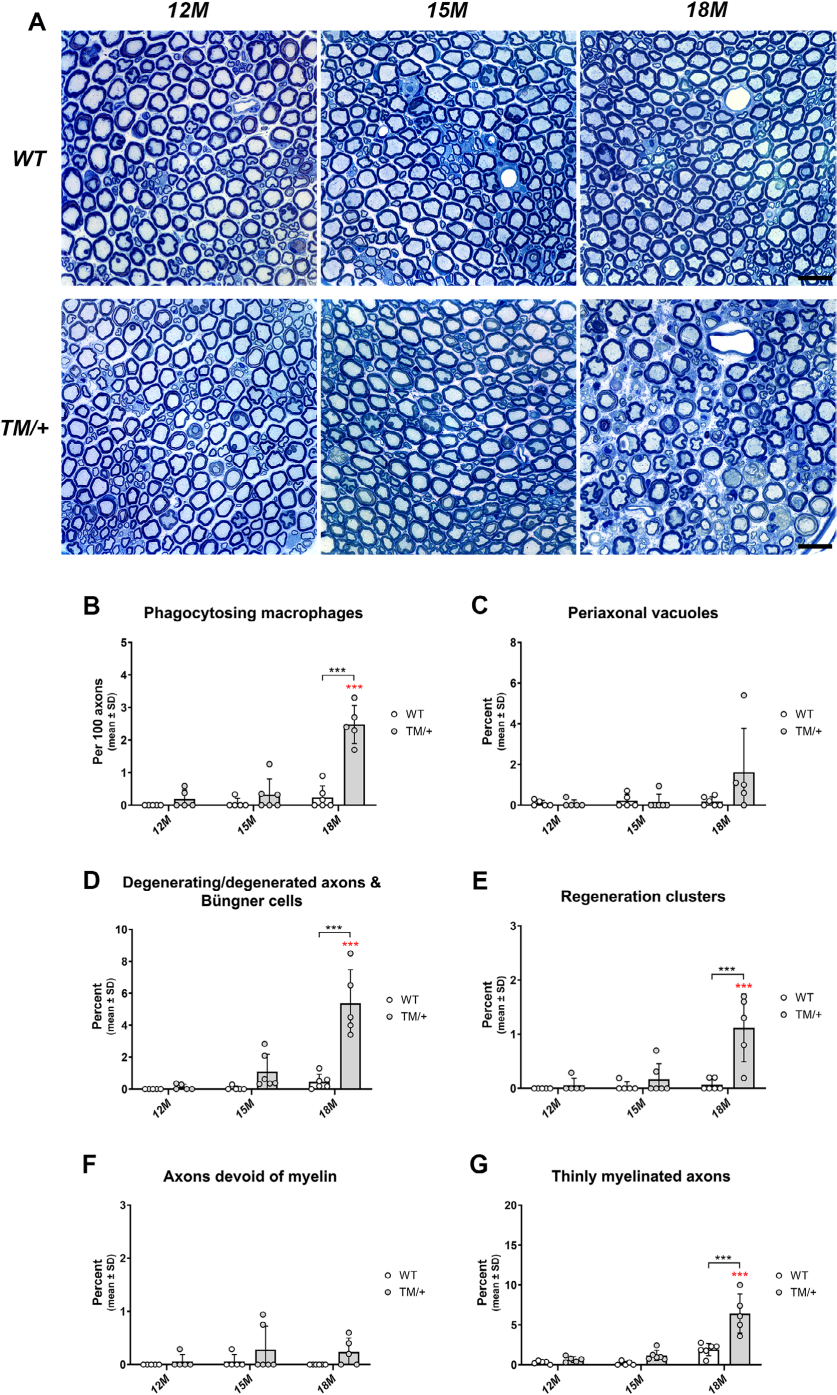
TM/+ mice develop a late‐onset axonopathy in distal femoral quadriceps nerves. (A) Representative semithin images of femoral quadriceps nerves of WT (upper panel) and TM/+ mice (lower panel) at 12 (left), 15 (middle) and 18 months of age (right). Note numerous axonopathic alterations (e.g., degenerating fibers) with only mild myelin abnormalities in 18‐month‐old TM/+ mice. Scale bar = 20 μm. (B) Quantification of phagocytosing macrophages reveals a significant increase in the frequency of phagocytosing macrophages in 18‐ compared to 12‐month‐old TM/+ mice (red asterisks) and also compared to age‐matched WT mice (black asterisks). (C–E) Quantification of typical axonopathic features demonstrates a non‐significant increase in the number of periaxonal vacuoles (C) in TM/+ compared to WT mice at 18 months of age. Signs of axon degeneration (D) and subsequent regeneration (E) appear in TM/+ mice starting at 15 months of age up to the significant increase at 18 months (red asterisks) and are, consequently, also significantly increased compared to age‐matched WT mice (black asterisks). (F, G) Quantification of axons devoid of myelin (F) and thinly myelinated axons (G) in WT and TM/+ mice. Axons devoid of myelin are scarce in both genotypes (F), whereas the prevalence of thinly myelinated fibers significantly increases in TM/+ mice at 18 months of age (G). In WT nerves, a slight but non‐significant increase in thinly myelinated axons is observed between 12 and 18 months of age. Note that phagocytosing macrophages and all other examined pathological alterations are barely detectable in WT femoral quadriceps nerves at any of the investigated time points. Two‐way ANOVA and Tukey's post hoc tests (B–G): ***p < 0.001.

**FIGURE 2:**
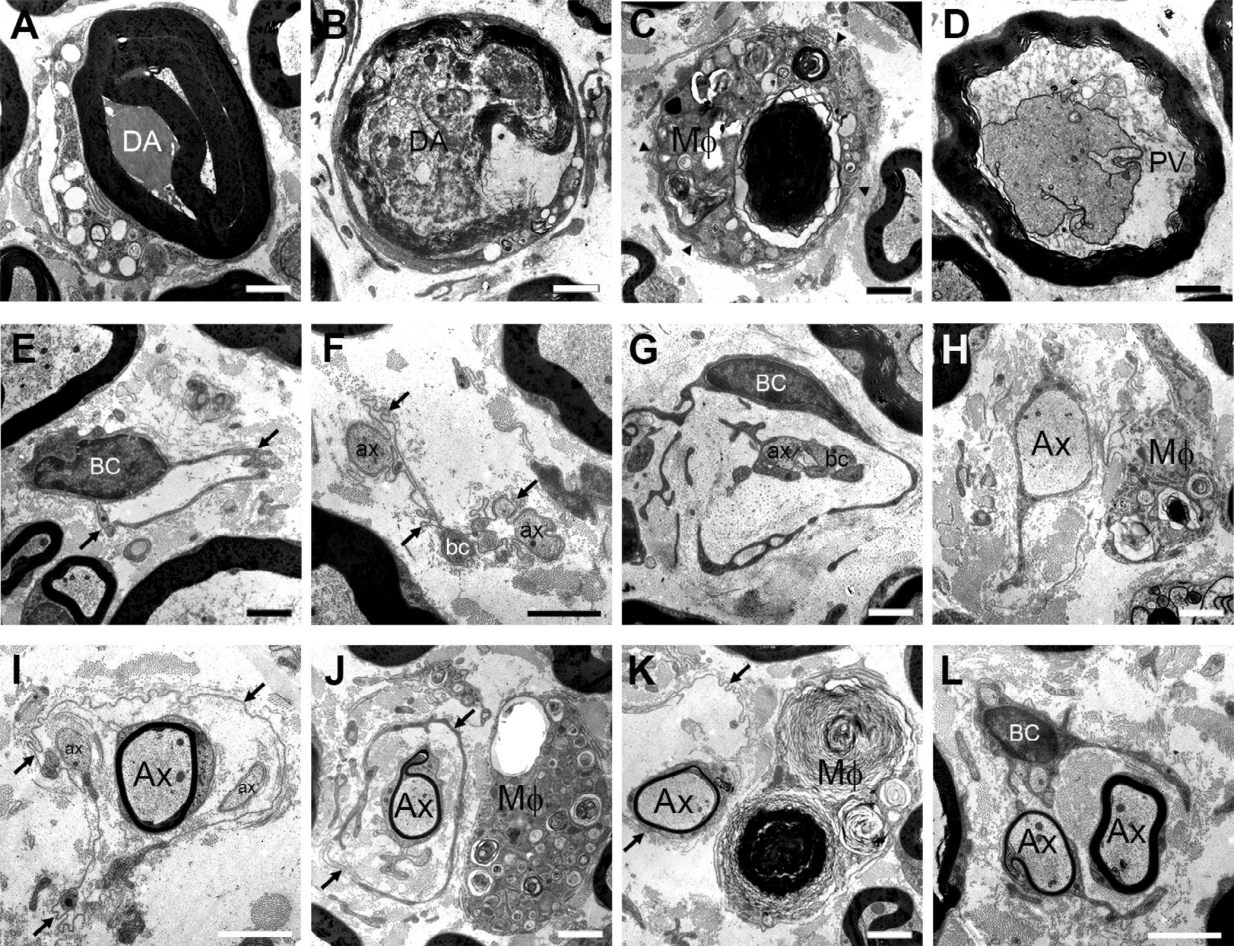
Axonopathic features in femoral quadriceps nerves of TM/+ mice. (A–C) Representative electron microscopical images of femoral quadriceps nerves of 18‐months‐old TM/+ mice demonstrating (A, B) degenerating axons (DA), and (C) myelin remnants of a degenerated axon cleared by a phagocytosing macrophage (Mϕ) that has penetrated the basal lamina (arrowheads). (D) Representative electron microscopical image of a periaxonal vacuole (PV). (E–G) Examples of Büngner cells (BC) formed after axonal degeneration and myelin clearance, with preservation of the basal lamina from the previously myelinating Schwann cell. Note Schwann cell processes (bc) associated with particularly small axonal sprouts (ax) within basal lamina tubes (arrows). (H–K) Axons devoid of myelin (H) and thinly myelinated axons (I–K) are predominantly associated with Büngner cells within the basal lamina tubes (arrows) and mainly consist of smaller‐caliber fibers (Ax), indicative of regenerating axons ensheathed by newly formed Schwann cell myelin. Note also the close proximity of activated/phagocytosing macrophages (Mϕ) with these fibers (H, J, K). (L) Representative electron microscopical image of regeneration clusters in nerves of TM/+ mice reflecting axon degeneration with subsequent regeneration. Scale bars (A–L) = 2 μm.

To determine the development of neuropathic alterations in the PNS of hemizygous P0T124M mutants (TM/+), we analyzed femoral quadriceps nerves in TM/+ mutant mice and WT littermates at 12, 15, and 18 months of age at light‐ and electron microscopical levels. Interestingly, TM/+ mice developed a severe axonopathy at 18 months of age (Figure 1A), closely reflecting observations from patient biopsies with the same genetic background (De Jonghe et al. 1999; Hanemann et al. 2001; Misu et al. 2000). This was accompanied by an increased presence of phagocytosing macrophages beginning at 15 months of age (Figure 1B, Figure 2A–C). Further morphological analysis at the ultrastructural level revealed an increase in typical axonopathic features like periaxonal vacuoles (Figures 1C and 2D), degenerating fibers (Figures 1D and 2A–C) or Büngner cells within basal lamina tubes (Figures 1D and 2E–G) together with regeneration clusters (Figures 1E and 2L) in TM/+ mutants. Also starting at 15 months of age, the frequency of axons devoid of myelin and thinly myelinated fibers was elevated in TM/+ mutants compared to WT littermates, reaching statistical significance at 18 months (Figure 1F,G). However, these pathological features differed from the typical demyelinating neuropathy phenotype, as they were predominantly associated with Büngner cells within the basal lamina tubes and mainly consisted of smaller‐caliber fibers (Figure 2H–K), indicative of regenerating axons undergoing Schwann cell remyelination (Helvacioglu and Dagdeviren 2019; Ide et al. 1990; Jortner 2020). Notably, the pathological alterations described above were absent in sensory saphenous nerves (data not shown).

**FIGURE 3:**
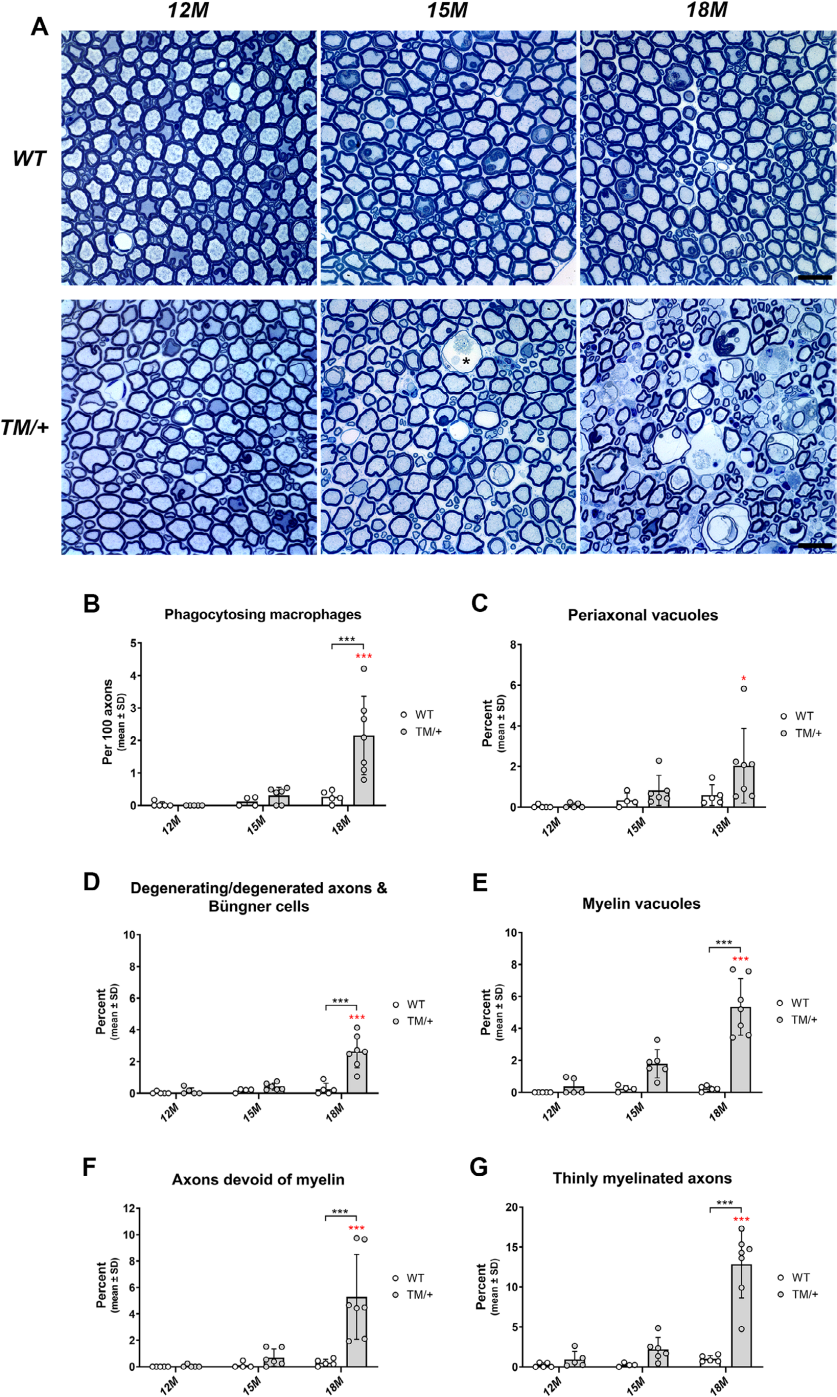
TM/+ mice develop a late‐onset axonopathy in lumbar ventral roots. (A) Representative semithin images of lumbar ventral roots of WT (upper panel) and TM/+ mice (lower panel) at 12 (left), 15 (middle) and 18 months of age (right). Note numerous axonopathic alterations (e.g., degenerating fibers) and myelin vacuoles in 18‐month‐old TM/+ mice. In TM/+ mice some macrophages are detectable in close proximity to axons within the periaxonal space; *, exemplarily shown for one representative profile). Scale bar = 20 μm. (B) Quantification of phagocytosing macrophages reveals a significant increase in the frequency of phagocytosing macrophages in 18‐ compared to 12‐month‐old TM/+ mice (red asterisks) and also compared to age‐matched WT mice (black asterisks). (C, D) Quantification of typical axonopathic features demonstrates a significant increase in the number of periaxonal vacuoles (C) and degenerating/degenerated fibers together with Büngner cells (D) in TM/+ compared to WT mice at 18 months of age. Signs of axon damage and degeneration appear in TM/+ mice starting at 15 months of age up to the significant increase at 18 months (red asterisks) and are, consequently, also significantly increased compared to age‐matched WT mice (black asterisks). (E) Quantification of myelin vacuoles reveals a progressive increase in the prevalence of this specific pathological sign in TM/+ mice during disease progression, reflected by significant changes between 18 and 12 months of age (red asterisks) and also significant elevation compared to age‐matched WT littermates (black asterisks). (F, G) Quantification of axons devoid of myelin (F) and thinly myelinated axons (G) demonstrates that the prevalence of these fibers with abnormal myelin significantly increases in TM/+ mice at 18 months of age. Note that phagocytosing macrophages and all examined pathological alterations are barely detectable in WT lumbar ventral roots at any of the investigated time points. Two‐way ANOVA and Tukey's post hoc tests: *p < 0.05; ***p < 0.001.

Next, we examined more proximal aspects of the peripheral nerves by analyzing lumbar ventral roots at the same time points. Consistent with our findings in femoral quadriceps nerves, axonopathic features and macrophage activation were detectable in lumbar ventral roots of TM/+ mice starting at 15 months of age and reaching statistical significance for all investigated parameters at 18 months (Figure 3). Interestingly, macrophages were also detectable in the periaxonal space, a feature not observed in femoral quadriceps nerves of TM/+ mice (Figure 3A). Furthermore, while regeneration clusters were rarely observed in lumbar ventral roots at the investigated time points (data not shown) myelin vacuoles were highly prevalent in TM/+ mice compared to their WT littermates (Figure 3A,E), contributing to a slightly earlier onset and more pronounced neuropathy in proximal compared to distal nerve aspects.

### 3.2 Late‐Onset Axonopathy in TM/+ Mice Is Associated With Increased Macrophage Numbers and Expression of Specific Activation Markers

**FIGURE 4:**
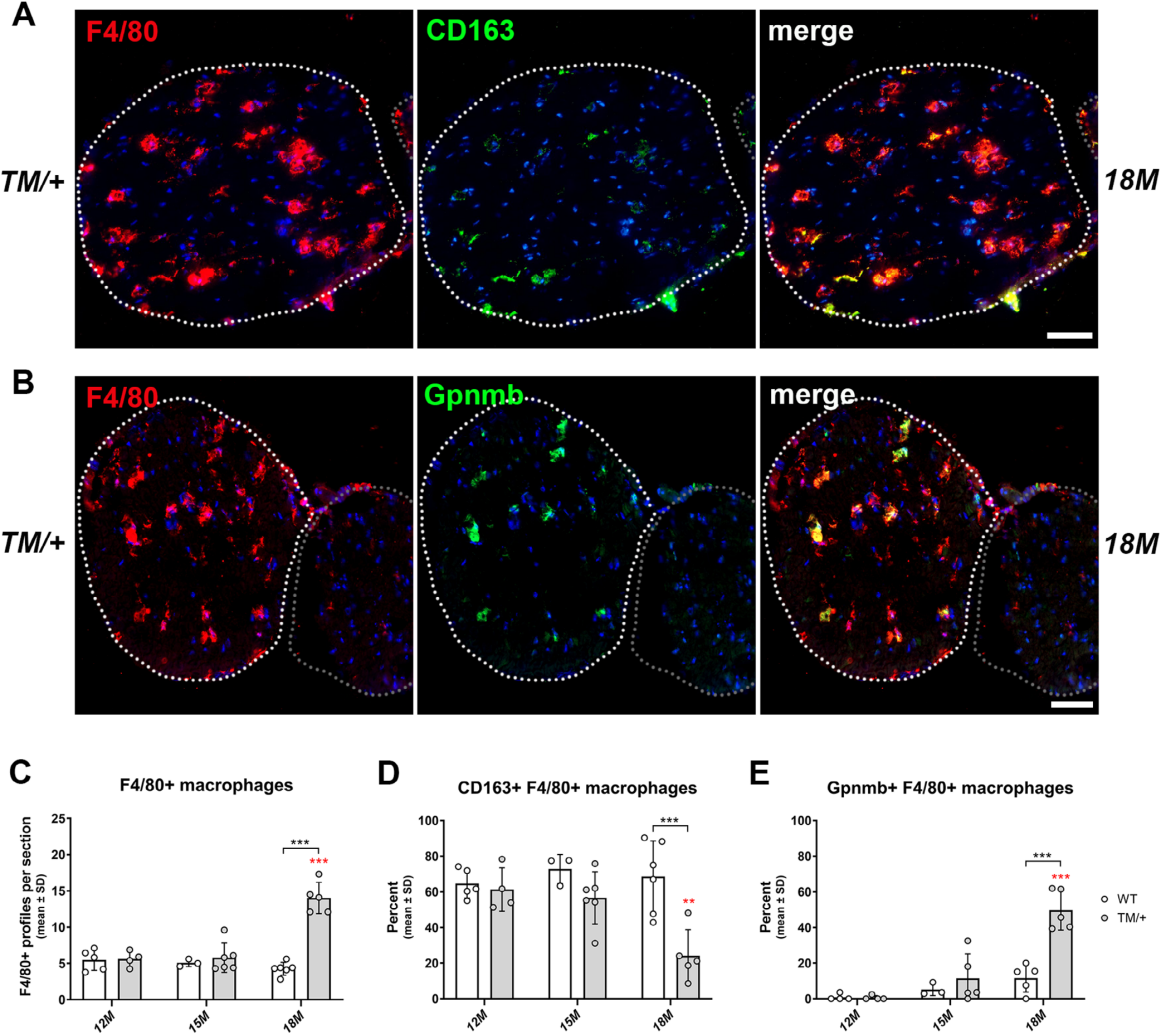
Late‐onset axonopathy in femoral quadriceps nerves is associated with the activation of macrophages in TM/+ mice. (A) Representative double‐immunohistochemical staining against the macrophage pan‐marker F4/80 (red) and the homeostatic marker CD163 (green) on cross‐sections of femoral nerves from 18‐month‐old TM/+ mice. White dashed circles represent the area of femoral quadriceps nerves, while gray dashed circles demarcate saphenous nerves. Nuclei are labeled with DAPI. Scale bar, 50 μm. (B) Representative double‐immunohistochemical staining against F4/80 (red) and the activation marker Gpnmb (green) on cross‐sections of femoral nerves from 18‐month‐old TM/+ mice. White dashed circles represent the area of femoral quadriceps nerves, while gray dashed circles demarcate saphenous nerves. Nuclei are labeled with DAPI. Scale bar, 50 μm. (C) Analysis of F4/80‐positive profiles in femoral quadriceps nerves reveals a significant increase in macrophage numbers in TM/+ mice between 12 and 18 months of age (red asterisks) whereas macrophage numbers remain stable in WT mice across the investigated time points. (D) Quantification of CD163‐expressing F4/80‐positive macrophages. The proportion of homeostatic macrophages in WT femoral quadriceps nerves remains constant (~65%) between 12 and 18 months of age, while TM/+ mice exhibit a significant decline in this macrophage subtype at 18 months. (E) Quantification of Gpnmb‐expressing F4/80‐positive macrophages. Activated macrophages are barely detectable in WT femoral quadriceps nerves but their proportion significantly increases in TM/+ mice at 18 months. Two‐way ANOVA and Tukey's post hoc tests: ***p < 0.001.

As phagocytosing macrophages were frequently detected in femoral quadriceps nerves and ventral roots of TM/+ mice, we further focused on macrophage numbers and their activation during disease progression by performing immunohistochemical stainings on cross‐sections of femoral quadriceps nerves. Quantifying the total number of macrophages with the pan‐macrophage marker F4/80 in WT and TM/+ mice revealed significant differences at 18 months of age (Figure 4A–C; Figure S1A,B).

Next, we determined the presence of homeostatic (CD163‐positive) and activated (Gpnmb‐positive) macrophages in femoral quadriceps nerves. Recently, *Cd163* was identified as a marker of tissue‐resident macrophages in naïve sciatic nerves, whereas elevated *Gpnmb* expression marked activated macrophages at the site of injury after peripheral nerve lesion (Zhao et al. 2022) and is also a characteristic feature of activated microglia observed in various neurodegenerative diseases, commonly referred to as “disease‐associated microglia” (DAM) (Huttenrauch et al. 2018; Song and Colonna 2018). Furthermore, these subtype‐specific markers were recently identified and validated among others as core signature genes of homeostatic and pathogenic macrophage clusters in the aging PNS (Klein et al. unpublished observations), where we previously demonstrated the pathological impact of macrophages (Yuan et al. 2018). Corroborating these findings, CD163‐expressing macrophages were highly abundant in WT mice at 12 months of age, while Gpnmb+ macrophages were almost absent in femoral quadriceps nerves (Figure 4; Figure S1). In WT mice, macrophages maintained their homeostatic state, with approximately 65% of F4/80‐positive macrophages consistently expressing CD163 across all examined time points (Figure 4D; Figure S1A). In contrast, TM/+ mice exhibited a significant decline in CD163/F4/80‐positive profiles between 12 and 18 months of age, suggesting a loss of homeostatic macrophages (Figure 4A,D). Reciprocally, Gpnmb‐expressing macrophages increased during disease progression in TM/+ mice (Figure 4B,E), whereas their numbers remained relatively low in age‐matched WT littermates (Figure 4E; Figure S1B).

In contrast to the findings in femoral quadriceps nerves, and consistent with the absence of neuropathy in sensory saphenous nerves (see above, data not shown), macrophage numbers did not increase in TM/+ mice compared to WT mice at any of the investigated time points (Figure S1C). Of note and also supporting our view of CD163 and Gpnmb as specific markers to determine macrophage activation, macrophages in saphenous nerves maintained their homeostatic phenotype, as indicated by the high abundance of CD163‐immuno reactivity and the absence of Gpnmb expression in F4/80‐positive macrophages (Figure S1D,E).

### 3.3 Treatment With PLX5622 Leads to a Robust Reduction in the Number of Macrophages but Does Not Alter Their Activation State

Using the established CSF‐1R inhibitor (CSF‐1Ri) PLX5622, we previously demonstrated its high efficacy in depleting macrophages in various CMT1 mouse models (Klein et al. 2022; Klein et al. 2015; Ostertag et al. 2022). Accordingly, and using the same protocols as in CMT1 models, we orally administered PLX5622 to TM/+ mice from 12 to 18 months of age, prior to the onset of obvious neuropathic alterations and the increase in macrophage numbers in peripheral nerves (Figure S2A).

**FIGURE 5:**
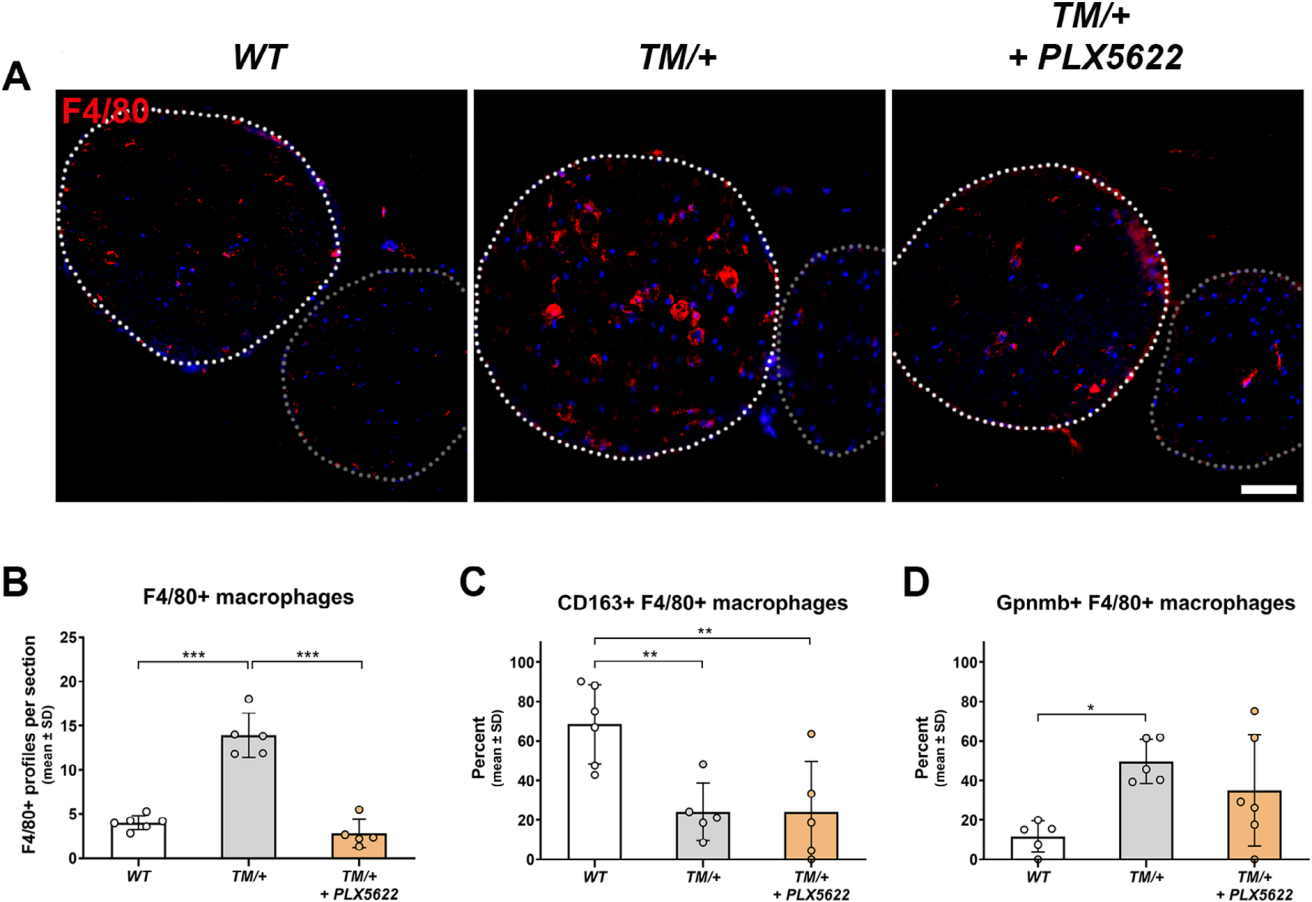
PLX5622 treatment reduces macrophage numbers in peripheral nerves of TM/+ mice. (A) Immunohistochemical staining against the macrophage pan‐marker F4/80 on cross‐sections of femoral nerves from 18‐month‐old WT (left), TM/+ (middle) and PLX5622‐treated TM/+ mice (right). White dashed circles represent the area of femoral quadriceps nerves, while gray dashed circles demarcate saphenous nerves. Nuclei are labeled with DAPI. Scale bar, 50 μm. (B) Corresponding quantification of F4/80‐positive profiles in femoral quadriceps nerves shows that the significant increase of macrophage numbers in TM/+ compared to WT mice is significantly prevented by PLX5622 treatment. (C) Quantification of the percentage of CD163‐expressing F4/80‐positive macrophages. PLX5622 treatment does not alter the proportion of homeostatic macrophages in femoral quadriceps nerves of TM/+ mice. (D) Quantification of the percentage of Gpnmb‐expressing F4/80‐positive macrophages. PLX5622 treatment does not alter the proportion of activated macrophages in femoral quadriceps nerves of TM/+ mice. One‐way ANOVA and Tukey's post hoc test: *p < 0.05; **p < 0.01; ***p < 0.001.

To analyze the effects of PLX5622 on the absolute number and the activation of macrophages, we performed immunohistochemical stainings on cross‐sections of femoral quadriceps nerves. Corroborating previous findings, PLX5622 treatment resulted in a robust depletion of macrophage numbers in femoral quadriceps and saphenous nerves (Figure 5A,B; Figure S2B). Interestingly, macrophages in saphenous nerves maintained their homeostatic phenotype (Figure S2C,D) and PLX5622 treatment did not significantly affect the elevated macrophage activation in femoral quadriceps nerves of TM/+ mutants, which was reflected by a similarly reduced expression of CD163 and increased presence of Gpnmb immunoreactivity in the remaining endoneurial macrophages (Figure 5C,D). These findings were also confirmed by immunohistochemical stainings against Mgl1 (also known as Clec10a; Figure S2E–G), another core signature gene associated with homeostatic macrophages in aging nerves (Klein et al. unpublished observation).

These findings indicate that PLX5622 treatment prevented the overall increase in macrophage numbers, while it did not affect their activation state in peripheral nerves of TM/+ mutant mice.

### 3.4 Targeting Macrophages With PLX5622 Prevents Distal Axon Degeneration in a Mouse Model for CMT2J

**FIGURE 6:**
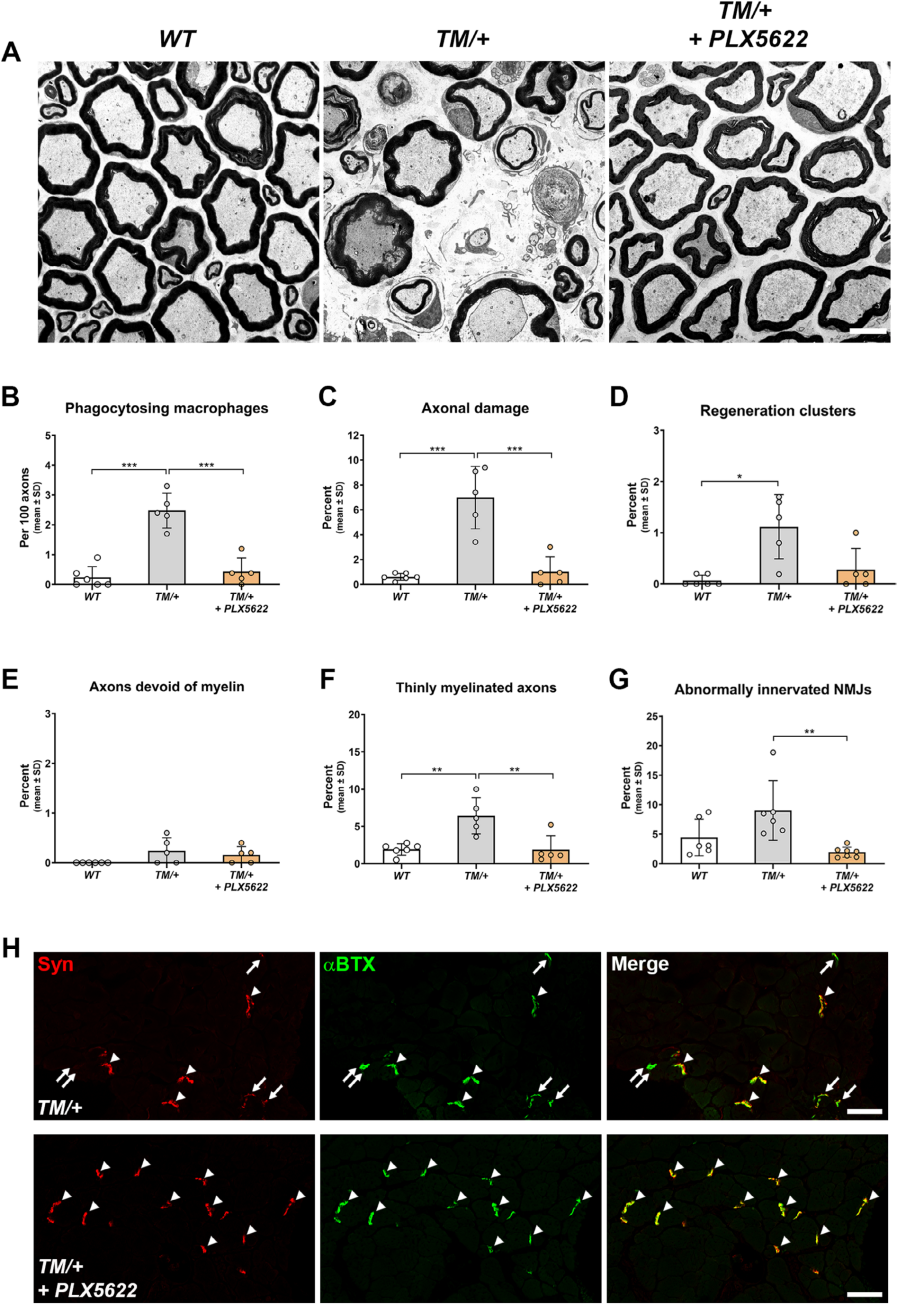
Targeting macrophages by PLX5622 prevents distal axonopathy in TM/+ mice. (A) Representative electron micrographs of femoral quadriceps nerves from 18‐month‐old WT (left), TM/+ (middle) and PLX5622‐treated TM/+ mice (right). The axonopathic phenotype in TM/+ mutant mice compared to WT littermates at 18 months of age is rescued by PLX5622 treatment. Scale bar: 5 μm. (B) Determination of phagocytosing macrophages reveals that PLX5622 treatment significantly reduces the frequency of this specific macrophage subtype in TM/+ mice. (C, D) Quantification of axonal damage, including periaxonal vacuoles, degenerating axons, and Büngner cells (C), as well as regeneration clusters (D), shows that PLX5622 treatment significantly prevents the increase in these axonopathic profiles in TM/+ mice, with a prevalence comparable to that observed in WT mice. (E, F) Quantification of axons devoid of myelin (E) and thinly myelinated axons (F) reveals significantly lower percentage of thinly myelinated fibers in PLX5622‐treated compared to untreated TM/+ mice. (G) Determination of abnormally innervated NMJs (comprising completely and partially denervated NMJs) from cross sections of flexor digitorum brevis (FDB) muscles demonstrates an increase of altered NMJs in TM/+ control compared to WT mice at 18 months of age. Abnormal muscle innervation is significantly prevented by PLX5622 treatment in TM/+ mice. One‐way ANOVA and Tukey's post hoc test (B, C, E–G), Kruskal‐Wallis and Dunn's multiple comparisons test (D): *p < 0.05; **p < 0.01; ***p < 0.001. (H) Examples of innervated (arrowheads), denervated (double arrows), and partially denervated NMJs (arrows) identified by double‐immunolabeling with synaptophysin (Syn, red) and α‐bungarotoxin (α BTX, green) in cross‐sections of FDB muscles from 18‐months‐old TM/+ control (top row) and PLX5622 treated TM/+ mice (bottom row). Scale bar = 50 μm.

After having demonstrated a significant reduction in the total numbers but not in the activation of macrophages following PLX5622 treatment in TM/+ mice, we examined histopathological changes at 18‐month‐old mice using electron microscopy. Remarkably, macrophage depletion prevented the development of neuropathy in femoral quadriceps nerves (Figure 6A). In addition to the decrease in phagocytosing macrophages (Figure 6B), axonopathic features were barely detectable in PLX5622‐treated TM/+ mice and comparable to WT reference values (Figure 6C,D). Correspondingly, axons devoid of myelin and thinly myelinated axons, likely representing axon regeneration and Schwann cell remyelination (Figure 2), were observed at a significantly lower frequency in treated TM/+ mice (Figure 6E,F). Subsequently, inhibiting axon degeneration through macrophage targeting significantly preserved distal NMJs innervation in TM/+ mice (Figure 6G,H).

**FIGURE 7:**
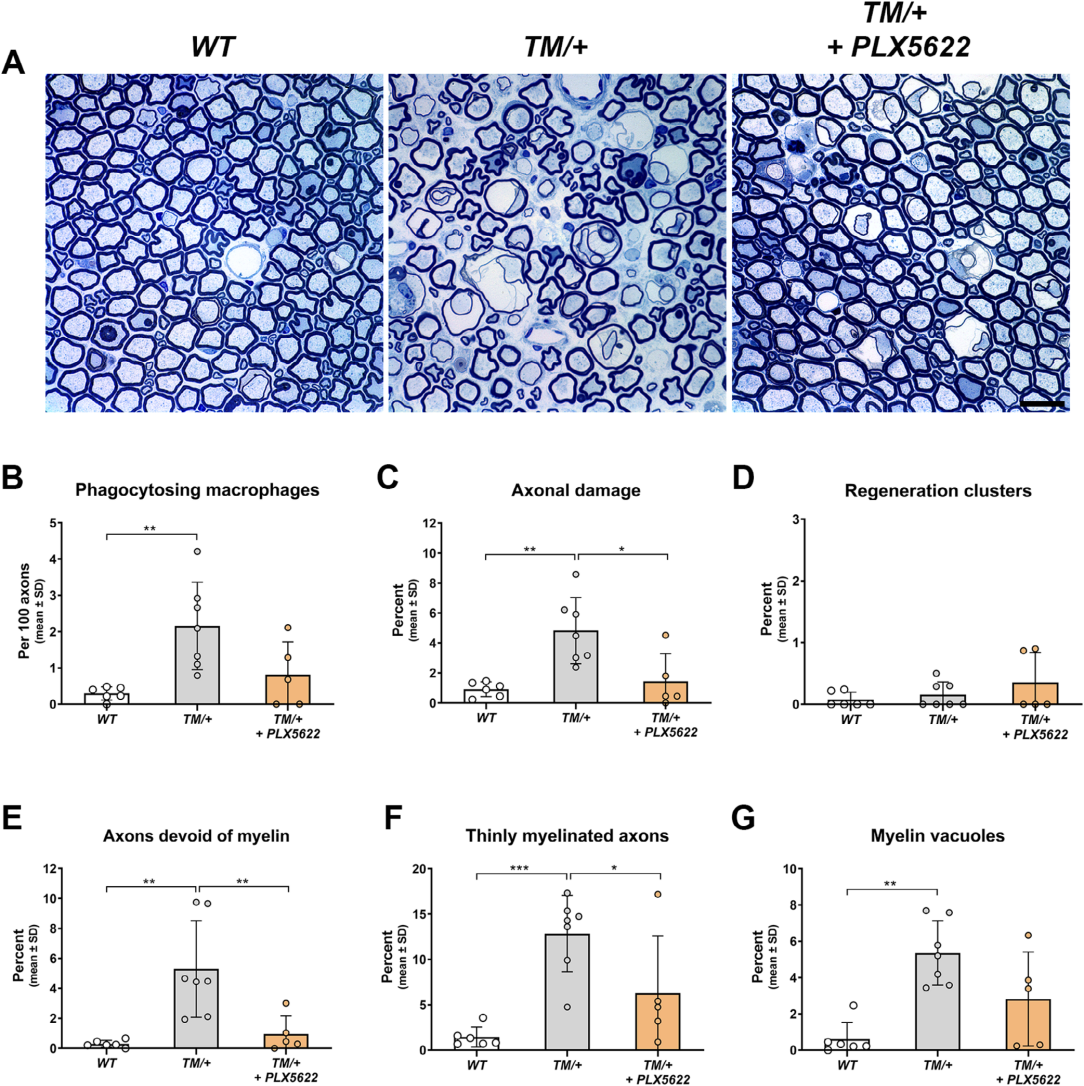
Targeting macrophages by PLX5622 mitigates axonopathic alterations in lumbar ventral roots of TM/+ mice. (A) Representative semithin sections of lumbar ventral roots from 18‐month‐old WT (left), TM/+ (middle) and PLX5622‐treated TM/+ mice (right). The axonopathic phenotype in TM/+ mutant mice compared to WT littermates at 18 months of age is robustly ameliorated by PLX5622 treatment. Scale bar, 20 μm. (B) Determination of phagocytosing macrophages reveals that PLX5622 treatment reduces the significant increase of this specific macrophage subtype in TM/+ mice. (C, D) Quantification of axonal damage, including periaxonal vacuoles, degenerating axons, and Büngner cells (C) shows that PLX5622 treatment significantly attenuates the prevalence of axonopathic profiles in TM/+ mice, with a prevalence comparable to that observed in WT mice. Note that regeneration clusters are, compared to distal nerves (Figure 6D), barely detectable in lumbar ventral roots at 18 months of age (D). (E, F) Quantification of axons devoid of myelin (E) and thinly myelinated axons (F) reveals significantly lower percentages of these abnormally myelinated fibers in PLX5622‐treated compared to untreated TM/+ mice. (G) Assessment of myelin vacuoles demonstrates a non‐significant reduction in these pathological profiles in TM/+ mice after PLX5622 treatment. One‐way ANOVA and Tukey's post hoc test (B, C, E, F), Kruskal‐Wallis and Dunn's multiple comparisons test (D, G): *p < 0.05; **p < 0.01; ***p < 0.001.

Next, focusing on proximal nerve aspects, morphological examination of lumbar ventral roots revealed that while PLX5622 treatment did not prevent the neuropathy and macrophage activation as robustly as in femoral nerves (Figure 7A,B), it significantly reduced axon damage and fibers with abnormal myelin in TM/+ mice (Figure 7C–F). Notably, myelin vacuoles were also observed at a lower frequency in PLX5622‐treated TM/+ mice (Figure 7G).

Our findings demonstrate that macrophage targeting had a substantial beneficial effect on disease outcome in the CMT2J mouse model, including a robust prevention of neuropathic features in femoral quadriceps nerves and muscle denervation.

### 3.5 Targeting Macrophages With PLX5622 Prevents the Functional Decline in a Mouse Model for CMT2J

**FIGURE 8:**
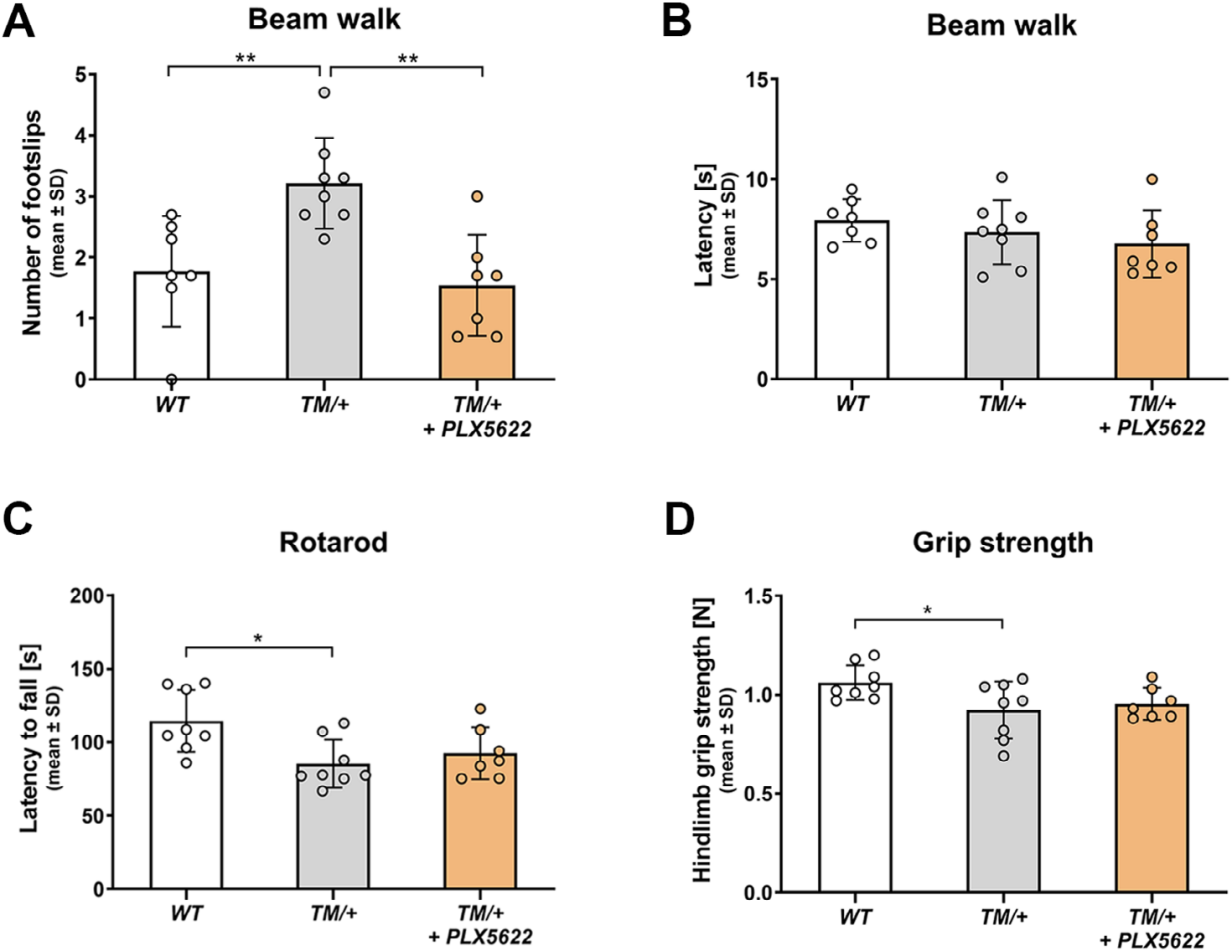
Targeting macrophages by PLX5622 leads to improved clinical outcome in TM/+ mice. (A) Analysis of the number of footslips in 18‐month‐old mice shows that TM/+ control mice exhibit a higher slip frequency while crossing the beam compared to WT mice. PLX5622 treatment prevents this functional decline in TM/+ mice. (B) The corresponding latency to cross the beam remains unchanged in TM/+ mice compared to WT littermates and is not affected by PLX5622 treatment. (C) Analysis of rotarod performance in 18‐month‐old mice demonstrates a significantly shorter latency to fall in TM/+ control mice compared to WT littermates. PLX5622 treatment mildly prolongs the latency to fall, leading to a slight improvement of motor coordination and balance in TM/+ mice. (D) Assessment of hind limb grip strength in 18‐month‐old mice reveals a significant reduction of strength in TM/+ control mice compared to WT littermates. PLX5622 treatment slightly attenuates the loss of hind limb grip strength in TM/+ mice. One‐way ANOVA and Tukey's post hoc test: *p < 0.05; **p < 0.01.

To evaluate the impact of PLX5622 treatment on motor performance in the CMT2J model, several clinical readout parameters were evaluated in 18‐month‐old mice. Beam walking tests indicated motor impairment in untreated TM/+ mice, as shown by a significantly higher number of foot slips (Figure 8A), while the latency to cross the beam remained unchanged between mutant and WT mice at this age (Figure 8B). Notably, PLX5622‐treated TM/+ mice exhibited fewer foot slips while crossing the beam compared to untreated TM/+ controls, with values similar to those of WT littermates (Figure 8A).

Measuring the latency to fall in the rotarod test also revealed significant deficits in untreated TM/+ mice compared to WT mice, while PLX5622 treatment resulted in a mild, non‐significant improvement (Figure 8C). Assessing hindlimb grip strength in mice showed comparable findings: while grip strength was reduced in TM/+ control compared to WT mice, PLX5622 treatment mildly ameliorated this significant decline (Figure 8D).

To further investigate nerve function, neurographic recordings were performed. Electrophysiological measurements revealed a slight decrease in NCV and mildly prolonged F‐wave latencies in 18‐month‐old TM/+ mice compared to WT littermates (Figure S3A,B). Surprisingly, despite the presence of axon degeneration, no significant decline in proximal or distal compound muscle action potentials (CMAPs) was observed in our hands in TM/+ mice (Figure S3C,D). Consequently, PLX5622‐treated TM/+ mutants showed no significant differences in neurographic recordings compared to the other experimental groups (Figure S3). While these findings are, at first glance, unexpected, previous studies by us and other groups also reported a similar discrepancy between nerve function and CMAP amplitudes (Fledrich et al. 2019; Kagiava et al. 2021; Ostertag et al. 2022; Prukop et al. 2019). It is conceivable that neurographic recordings primarily reflect sciatic nerve function, which, in 18‐month‐old TM/+ mice, exhibits a milder pathological phenotype compared to the femoral quadriceps nerve and ventral roots (Klein et al. unpublished observations and Shackleford et al. 2022).

Despite this observation, our results suggest that targeting macrophages with PLX5622 effectively prevented the decline of clinical parameters that were observed to be deteriorating in untreated 18‐month‐old TM/+ mice, highlighting a potential neuroprotective role of this treatment.

## 4 Discussion

While neuroinflammation is well‐documented as a pathogenic mediator in demyelinating CMT1 models, its role in axonal CMT2 subtypes remains less clear (Bolino and D'Antonio 2023; Kaminska and Kochanski 2024; Klein and Martini 2016; Martini et al. 2013). Studies in *Gdap1*‐deficient mice suggest that an inflammatory response in peripheral nerves implicating activated macrophages might be involved in axonal degeneration of the corresponding CMT2K model (Fernandez‐Lizarbe et al. 2019). However, the pathological implications of macrophage activation in affected peripheral nerves have not yet been systematically explored in axonal CMT forms.

A recent mouse model for another axonal CMT subtype, CMT2J, was generated by substituting threonine with methionine at position 124 of P0 (P0T124M). Notably, several pathological features characteristic of axonal CMT were observed in peripheral nerves (Shackleford et al. 2022). The presence of activated macrophages further suggests a potential role of neuroinflammation in disease progression. These initial studies primarily focused on homozygous P0T124M mutants (TM/TM; Shackleford et al. 2022) which develop an early‐onset neuropathy in femoral quadriceps nerves, characterized by rapid disease progression (Klein, Martini unpublished observations; Claessens and D'Antonio unpublished observations) and resembling nerve biopsy findings in a rare homozygous patient (Kurihara et al. 2003). Since the vast majority of CMT2J patients carry only one mutated allele and develop a late‐onset neuropathy (Kurihara et al. 2003; Misu et al. 2000), we now performed a detailed analysis in hemizygous P0T124M mutant mice (TM/+) to investigate axonopathic alterations during disease progression and, furthermore, to explore the role of macrophages in axonal CMT2.

Here we show that TM/+ mice develop a late‐onset axonopathy that closely resembles the neuropathy observed in corresponding CMT2J patients (De Jonghe et al. 1999; Hanemann et al. 2001; Misu et al. 2000). At 18 months of age, femoral quadriceps nerves and lumbar ventral roots of TM/+ mice display axonal degeneration, leading to muscle denervation and subsequent functional impairment. Interestingly, pathological alterations were absent in sensory saphenous nerves (data not shown), which is in line with findings in *Mpz* heterozygous knockout (P0het) mice (Martini 1997) where a mild and progressive neuropathy develops only in motor nerves and distal sensory nerves remain preserved (Giese et al. 1992; Martini et al. 1995). Notably, degenerative features in TM/+ mice were associated with a reduction in homeostatic (CD163^+^; Mgl1^+^) macrophages and a marked increase in activated (Gpnmb^+^) macrophages, suggesting a potential role for activated macrophages in CMT2J disease progression.

To investigate the therapeutic potential of macrophage targeting in axonal CMT2J, we administered the CSF‐1R inhibitor PLX5622 from 12 to 18 months of age. Corroborating previous studies in CMT1 mouse models (Klein et al. 2022; Klein et al. 2015; Ostertag et al. 2022) this treatment effectively reduced the total number of macrophages in peripheral nerves of TM/+ mice. Remarkably, macrophage targeting before disease onset (i.e., 12 months) prevented primary axonal degeneration and blocked the development of typical neuropathic features in distal peripheral nerves at 18 months of age. Eventually, PLX5622 preserved muscle denervation and mitigated some parameters of clinical impairment.

Our findings highlight the critical role of macrophages in the progression of axonal degeneration in the CMT2J mouse model and support the potential of macrophage‐targeting therapies in related peripheral neuropathies. Notably, compared to previous studies on PLX5622 treatment in demyelinating CMT models (Klein et al. 2022; Klein et al. 2015; Ostertag et al. 2022), the beneficial effects observed in this axonal model were even more pronounced as it prevented the development of the neuropathy in distal nerves.

Interestingly, a recent study also demonstrated that macrophage depletion prevented primary axon degeneration in a motor‐nerve‐dependent SARMopathy, highlighting a SARM1‐dependent neuroimmune mechanism as a key driver of disease progression (Dingwall et al. 2022). Similar protective effects upon macrophage targeting suggest comparable pathogenic roles of macrophages in SARM1‐mutant mice. As a reciprocal approach, future experiments should also investigate SARM1 activation in hemizygous TM/+ mice and its role in macrophage‐mediated axonal damage.

How does a mutation in the glial protein MPZ lead to a late‐onset axonopathy in mice and men (Li et al. 2006; Moss et al. 2021; Sanmaneechai et al. 2015; Shy et al. 2004)? It is reasonable to suggest that the respective MPZ mutations disrupt glia–axon communication, leading to axonal perturbation (Nave 2010a, 2010b). This could lead to secondary inflammatory reactions reminiscent of Wallerian degeneration. Wallerian degeneration and CMT share similar mechanisms regarding the activation of innate immune cells. After nerve injury, axons initially degenerate, regulating the recruitment and activation of macrophages via Schwann cell‐secreted cytokines, eventually clearing degenerating axons and myelin (Martini et al. 2008; Martini et al. 2013). If similar mechanisms implicating the Schwann cell‐related MEK/ERK‐CCL2 signaling pathway are also activated in TM/+ mice, that might contribute to macrophage recruitment and nerve damage as in distinct demyelinating CMT1 models (Fischer et al. 2008; Groh et al. 2010; Kohl et al. 2010) need to be analyzed in future studies.

Unlike Wallerian degeneration, the prevention of axonopathy in TM/+ mice upon macrophage targeting with PLX5622 strongly suggests that macrophages actively perturb axons in CMT2J. One possibility is that macrophages directly damage axons through the release of pro‐inflammatory cytokines, such as TNF‐α, or other neurotoxic mediators, as described in inflammatory neuropathies (Kiefer et al. 2001). As macrophages are frequently observed in close proximity to mostly intact‐appearing axons in lumbar ventral roots of TM/+ mice, it is tempting to speculate that they may also target axons by infiltrating into the periaxonal space and disturbing axon‐glial organization, a mechanism previously demonstrated in acute motor axonal neuropathy (AMAN) (Griffin et al. 1996; Hafer‐Macko et al. 1996; Martini and Willison 2016). In addition, recent findings suggest that stressed but viable axons expose phosphatidylserine (PS), thereby fostering their phagocytic discoverability and removal (Dingwall et al. 2022). Indirect mechanisms eventually leading to secondary axon degeneration in TM/+ mice are also conceivable: macrophages may target perturbed myelinating Schwann cells, resulting in disrupted axon‐glia signaling, impaired metabolic support, or compromised axonal transport (Boucanova and Chrast 2020; Deck et al. 2022). Importantly, macrophage‐induced structural and functional changes of glial cells leading to axon perturbation are also conceivable. For instance, in the CNS of mice carrying pathogenic PLP mutations, cytotoxic T‐lymphocytes target mutant glial cells, leading to oligodendrocyte‐mediated axonal constriction and damage at the paranodal domains (Groh et al. 2023). It is plausible to hypothesize that basal lamina‐penetrating macrophages, as observed in ventral spinal roots of TM/+ mice, might trigger similar detrimental axon‐directed reactions of mutant Schwann cells, an option which should be a matter of future studies.

Interestingly, our findings suggest that ventral roots in TM/+ are affected slightly earlier than peripheral nerves and may involve distinct underlying disease mechanisms. While especially pronounced myelin vacuolization was observed in ventral roots, this pathological feature was barely detectable in quadriceps nerves. Additionally, proximal nerve segments exhibited limited regenerative capacity, whereas distal nerves showed a significantly higher prevalence of regeneration clusters, indicating distinct responses to axon degeneration. To further investigate these differences, transcriptomic studies of myeloid and glial cells in differently affected nerves (e.g., femoral quadriceps nerves and ventral roots) could provide valuable insights into distinct pathogenic pathways in different nerves and region‐specific degeneration mechanisms in peripheral neuropathies. Furthermore, those studies would be crucial to define the molecular signature of nerve macrophages (Amann and Prinz 2020; Hakim et al. 2025; Wang et al. 2020; Ydens et al. 2020) and investigate whether similar or distinct macrophage populations contribute to the pathology in axonal versus demyelinating CMT neuropathies. A more comprehensive understanding of disease‐specific inflammatory mechanisms is essential to characterize the pathogenic state of nerve macrophages and develop future therapeutic approaches that selectively target pathogenic macrophages.

In summary, we demonstrate for the first time that neuroinflammation contributes to disease progression in a late‐onset axonal CMT neuropathy and that macrophage targeting represents a promising therapeutic approach for CMT2J. Future studies should explore whether macrophages also actively contribute to the pathology of other CMT2 subtypes associated with non‐glial mutations, such as CMT2A (*Mfn2*) or CMT2E (*Nefl*), potentially revealing a common pathogenic mechanism across distinct CMT forms and uncovering shared therapeutic targets for treatment.

## Author Contributions

D.K. and R.M. designed the research. D.K., N.E., X.Y. performed the research. D.K., N.E., and R.M. analyzed the data. G.G.S., A.C., M.L.F., L.W., and M.D.'A. generated and provided P0T12M mice. D.K. and R.M. wrote the manuscript with input from all authors.

## Conflicts of Interest

The authors declare no conflicts of interest.

## Supporting information


**Data S1:** Supporting Information.

**Figure S1:** Macrophages in sensory saphenous nerves of TM/+ mice do not increase in number and retain their homeostatic phenotype.

**Figure S2:** PLX5622 treatment effects on macrophages in sensory saphenous nerves.

**Figure S3:** Electrophysiological recordings do not show significant alterations in TM/+ mutants compared to age‐matched WT mice.

## Data Availability

The data that support the findings of this study are available from the corresponding author upon reasonable request.

## References

[glia70074-bib-0001] Amann, L. , and M. Prinz . 2020. “The Origin, Fate and Function of Macrophages in the Peripheral Nervous System—An Update.” International Immunology 32, no. 11: 709–717. 10.1093/intimm/dxaa030. 32322888

[glia70074-bib-0002] Berghoff, M. , M. Samsam , M. Muller , et al. 2005. “Neuroprotective Effect of the Immune System in a Mouse Model of Severe Dysmyelinating Hereditary Neuropathy: Enhanced Axonal Degeneration Following Disruption of the RAG‐1 Gene.” Molecular and Cellular Neurosciences 28, no. 1: 118–127. 15607947 10.1016/j.mcn.2004.09.001

[glia70074-bib-0003] Bolino, A. , and M. D'Antonio . 2023. “Recent Advances in the Treatment of Charcot‐Marie‐Tooth Neuropathies.” Journal of the Peripheral Nervous System 28, no. 2: 134–149. 10.1111/jns.12539. 36855793

[glia70074-bib-0004] Boucanova, F. , and R. Chrast . 2020. “Metabolic Interaction Between Schwann Cells and Axons Under Physiological and Disease Conditions.” Frontiers in Cellular Neuroscience 14: 148. 10.3389/fncel.2020.00148. 32547370 PMC7274022

[glia70074-bib-0005] Callegari, I. , C. Gemelli , A. Geroldi , et al. 2019. “Mutation Update for Myelin Protein Zero‐Related Neuropathies and the Increasing Role of Variants Causing a Late‐Onset Phenotype.” Journal of Neurology 266, no. 11: 2629–2645. 10.1007/s00415-019-09453-3. 31278453

[glia70074-bib-0006] Chapon, F. , P. Latour , P. Diraison , S. Schaeffer , and A. Vandenberghe . 1999. “Axonal Phenotype of Charcot‐Marie‐Tooth Disease Associated With a Mutation in the Myelin Protein Zero Gene.” Journal of Neurology, Neurosurgery, and Psychiatry 66, no. 6: 779–782. 10.1136/jnnp.66.6.779. 10329755 PMC1736388

[glia70074-bib-0007] De Jonghe, P. , V. Timmerman , C. Ceuterick , et al. 1999. “The Thr124Met Mutation in the Peripheral Myelin Protein Zero (MPZ) Gene Is Associated With a Clinically Distinct Charcot‐Marie‐Tooth Phenotype.” Brain 122, no. Pt 2: 281–290. 10.1093/brain/122.2.281. 10071056

[glia70074-bib-0008] Deck, M. , G. Van Hameren , G. Campbell , et al. 2022. “Physiology of PNS Axons Relies on Glycolytic Metabolism in Myelinating Schwann Cells.” PLoS One 17, no. 10: e0272097. 10.1371/journal.pone.0272097. 36194565 PMC9531822

[glia70074-bib-0009] Dingwall, C. B. , A. Strickland , S. W. Yum , et al. 2022. “Macrophage Depletion Blocks Congenital SARM1‐Dependent Neuropathy.” bioRxiv, 2022.2002.2026.482110. 10.1101/2022.02.26.482110. PMC9711884 36287209

[glia70074-bib-0010] Ey, B. , I. Kobsar , H. Blazyca , A. Kroner , and R. Martini . 2007. “Visualization of Degenerating Axons in a Dysmyelinating Mouse Mutant With Axonal Loss.” Molecular and Cellular Neurosciences 35, no. 1: 153–160. 10.1016/j.mcn.2007.02.014. 17383197

[glia70074-bib-0011] Faul, F. , E. Erdfelder , A. G. Lang , and A. Buchner . 2007. “G*Power 3: A Flexible Statistical Power Analysis Program for the Social, Behavioral, and Biomedical Sciences.” Behavior Research Methods 39, no. 2: 175–191. 10.3758/Bf03193146. 17695343

[glia70074-bib-0012] Fernandez‐Lizarbe, S. , A. Civera‐Tregon , L. Cantarero , et al. 2019. “Neuroinflammation in the Pathogenesis of Axonal Charcot‐Marie‐Tooth Disease Caused by Lack of GDAP1.” Experimental Neurology 320: 113004. 10.1016/j.expneurol.2019.113004. 31271761

[glia70074-bib-0013] Fischer, S. , C. Kleinschnitz , M. Müller , et al. 2008. “Monocyte Chemoattractant Protein‐1 Is a Pathogenic Component in a Model for a Hereditary Peripheral Neuropathy.” Molecular and Cellular Neurosciences 37: 359–366. 18326085 10.1016/j.mcn.2007.10.012

[glia70074-bib-0014] Fledrich, R. , D. Akkermann , V. Schutza , et al. 2019. “NRG1 Type I Dependent Autoparacrine Stimulation of Schwann Cells in Onion Bulbs of Peripheral Neuropathies.” Nature Communications 10, no. 1: 1467. 10.1038/s41467-019-09385-6. PMC6443727 30931926

[glia70074-bib-0015] Giese, K. P. , R. Martini , G. Lemke , P. Soriano , and M. Schachner . 1992. “Mouse P0 Gene Disruption Leads to Hypomyelination, Abnormal Expression of Recognition Molecules, and Degeneration of Myelin and Axons.” Cell 71, no. 4: 565–576. 1384988 10.1016/0092-8674(92)90591-y

[glia70074-bib-0016] Griffin, J. W. , C. Y. Li , C. Macko , et al. 1996. “Early Nodal Changes in the Acute Motor Axonal Neuropathy Pattern of the Guillain‐Barre Syndrome.” Journal of Neurocytology 25, no. 1: 33–51. 8852937 10.1007/BF02284784

[glia70074-bib-0017] Groh, J. , T. Abdelwahab , Y. Kattimani , et al. 2023. “Microglia‐Mediated Demyelination Protects Against CD8(+) T Cell‐Driven Axon Degeneration in Mice Carrying PLP Defects.” Nature Communications 14, no. 1: 6911. 10.1038/s41467-023-42570-2. PMC10616105 37903797

[glia70074-bib-0018] Groh, J. , R. Basu , E. R. Stanley , and R. Martini . 2016. “Cell‐Surface and Secreted Isoforms of CSF‐1 Exert Opposing Roles in Macrophage‐Mediated Neural Damage in Cx32‐Deficient Mice.” Journal of Neuroscience: The Official Journal of the Society for Neuroscience 36, no. 6: 1890–1901. 10.1523/JNEUROSCI.3427-15.2016. 26865613 PMC4748074

[glia70074-bib-0019] Groh, J. , K. Heinl , B. Kohl , et al. 2010. “Attenuation of MCP‐1/CCL2 Expression Ameliorates Neuropathy in a Mouse Model for Charcot‐Marie‐Tooth 1X.” Human Molecular Genetics 19, no. 18: 3530–3543. 20591826 10.1093/hmg/ddq269

[glia70074-bib-0020] Groh, J. , J. Weis , H. Zieger , E. R. Stanley , H. Heuer , and R. Martini . 2012. “Colony‐Stimulating Factor‐1 Mediates Macrophage‐Related Neural Damage in a Model for Charcot‐Marie‐Tooth Disease Type 1X.” Brain: A Journal of Neurology 135, no. Pt 1: 88–104. 10.1093/brain/awr283. 22094537 PMC3267979

[glia70074-bib-0021] Hafer‐Macko, C. , S. T. Hsieh , C. Y. Li , et al. 1996. “Acute Motor Axonal Neuropathy: An Antibody‐Mediated Attack on Axolemma.” Annals of Neurology 40, no. 4: 635–644. 10.1002/ana.410400414. 8871584

[glia70074-bib-0022] Hakim, S. , A. Jain , S. S. Adamson , et al. 2025. “Macrophages Protect Against Sensory Axon Loss in Peripheral Neuropathy.” Nature 640, no. 8057: 212–220. 10.1038/s41586-024-08535-1. 39939762 PMC11964918

[glia70074-bib-0023] Hanemann, C. O. , A. A. Gabreels‐Festen , and P. De Jonghe . 2001. “Axon Damage in CMT due to Mutation in Myelin Protein P0.” Neuromuscular Disorders 11, no. 8: 753–756. 10.1016/s0960-8966(01)00229-2. 11595518

[glia70074-bib-0024] Helvacioglu, F. , and A. Dagdeviren . 2019. “Myelin Ultrastructure Terminology in Disease and Recovery Processes.” Archives Italiennes de Biologie 157, no. 2–3: 76–88. 10.12871/00039829201924. 31821531

[glia70074-bib-0025] Huttenrauch, M. , I. Ogorek , H. Klafki , et al. 2018. “Glycoprotein NMB: A Novel Alzheimer's Disease Associated Marker Expressed in a Subset of Activated Microglia.” Acta Neuropathologica Communications 6, no. 1: 108. 10.1186/s40478-018-0612-3. 30340518 PMC6194687

[glia70074-bib-0026] Ide, C. , T. Osawa , and K. Tohyama . 1990. “Nerve Regeneration Through Allogeneic Nerve Grafts, With Special Reference to the Role of the Schwann Cell Basal Lamina.” Progress in Neurobiology 34, no. 1: 1–38. 10.1016/0301-0082(90)90024-b. 2406794

[glia70074-bib-0027] Jerath, N. U. , and M. E. Shy . 2015. “Hereditary Motor and Sensory Neuropathies: Understanding Molecular Pathogenesis Could Lead to Future Treatment Strategies.” Biochimica et Biophysica Acta 1852, no. 4: 667–678. 10.1016/j.bbadis.2014.07.031. 25108281

[glia70074-bib-0028] Jortner, B. S. 2020. “Nerve Fiber Regeneration in Toxic Peripheral Neuropathy.” Toxicologic Pathology 48, no. 1: 144–151. 10.1177/0192623319854089. 31184283

[glia70074-bib-0029] Kagiava, A. , C. Karaiskos , J. Richter , et al. 2021. “AAV9‐Mediated Schwann Cell‐Targeted Gene Therapy Rescues a Model of Demyelinating Neuropathy.” Gene Therapy 28: 659–675. 10.1038/s41434-021-00250-0. 33692503 PMC8599011

[glia70074-bib-0030] Kaminska, J. , and A. Kochanski . 2024. “A Role of Inflammation in Charcot‐Marie‐Tooth Disorders‐In a Perspective of Treatment?” International Journal of Molecular Sciences 26, no. 1. 10.3390/ijms26010015. PMC11720021 39795872

[glia70074-bib-0031] Kiefer, R. , B. C. Kieseier , G. Stoll , and H. P. Hartung . 2001. “The Role of Macrophages in Immune‐Mediated Damage to the Peripheral Nervous System.” Progress in Neurobiology 64, no. 2: 109–127. 11240209 10.1016/s0301-0082(00)00060-5

[glia70074-bib-0032] Klein, D. , J. Groh , X. Yuan , et al. 2022. “Early Targeting of Endoneurial Macrophages Alleviates the Neuropathy and Affects Abnormal Schwann Cell Differentiation in a Mouse Model of Charcot‐Marie‐Tooth 1A.” Glia 70: 1100–1116. 10.1002/glia.24158. 35188681

[glia70074-bib-0033] Klein, D. , and R. Martini . 2016. “Myelin and Macrophages in the PNS: An Intimate Relationship in Trauma and Disease.” Brain Research 1641, no. Pt A: 130–138. 10.1016/j.brainres.2015.11.033. 26631844

[glia70074-bib-0034] Klein, D. , A. Patzko , D. Schreiber , et al. 2015. “Targeting the Colony Stimulating Factor 1 Receptor Alleviates Two Forms of Charcot‐Marie‐Tooth Disease in Mice.” Brain: A Journal of Neurology 138, no. Pt 11: 3193–3205. 10.1093/brain/awv240. 26297559

[glia70074-bib-0035] Klein, D. , M. G. Yepez , and R. Martini . 2024. “Physical Exercise Halts Further Functional Decline in an Animal Model for Charcot‐Marie‐Tooth Disease 1X at an Advanced Disease Stage.” Journal of the Peripheral Nervous System 29: 494–504. 10.1111/jns.12669. 39523026 PMC11625978

[glia70074-bib-0036] Klein, D. , X. Yuan , E. M. Weiss , and R. Martini . 2021. “Physical Exercise Mitigates Neuropathic Changes in an Animal Model for Charcot‐Marie‐Tooth Disease 1X.” Experimental Neurology 343: 113786. 10.1016/j.expneurol.2021.113786. 34153322

[glia70074-bib-0037] Kohl, B. , S. Fischer , J. Groh , C. Wessig , and R. Martini . 2010. “MCP‐1/CCL2 Modifies Axon Properties in a PMP22‐Overexpressing Mouse Model for Charcot‐Marie‐Tooth 1A Neuropathy.” American Journal of Pathology 176, no. 3: 1390–1399. 20093502 10.2353/ajpath.2010.090694 PMC2832158

[glia70074-bib-0038] Kurihara, S. , Y. Adachi , K. Wada , A. Adachi , E. Ohama , and K. Nakashima . 2003. “Axonal and Demyelinating Forms of the MPZ Thr124Met Mutation.” Acta Neurologica Scandinavica 108, no. 3: 157–160. 10.1034/j.1600-0404.2003.00110.x. 12911457

[glia70074-bib-0039] Li, J. , Y. Bai , E. Ianakova , et al. 2006. “Major Myelin Protein Gene (P0) Mutation Causes a Novel Form of Axonal Degeneration.” Journal of Comparative Neurology 498, no. 2: 252–265. 10.1002/cne.21051. 16856127

[glia70074-bib-0040] Martini, R. 1997. “Animal Models for Inherited Peripheral Neuropathies.” Journal of Anatomy 191: 321–336. 9418989 10.1046/j.1469-7580.1997.19130321.x PMC1467690

[glia70074-bib-0041] Martini, R. , S. Fischer , R. Lopez‐Vales , and S. David . 2008. “Interactions Between Schwann Cells and Macrophages in Injury and Inherited Demyelinating Disease.” Glia 56, no. 14: 1566–1577. 10.1002/glia.20766. 18803324

[glia70074-bib-0042] Martini, R. , D. Klein , and J. Groh . 2013. “Similarities Between Inherited Demyelinating Neuropathies and Wallerian Degeneration: An Old Repair Program May Cause Myelin and Axon Perturbation Under Nonlesion Conditions.” American Journal of Pathology 183, no. 3: 655–660. 10.1016/j.ajpath.2013.06.002. 23831295

[glia70074-bib-0043] Martini, R. , and H. Willison . 2016. “Neuroinflammation in the Peripheral Nerve: Cause, Modulator, or Bystander in Peripheral Neuropathies?” Glia 64, no. 4: 475–486. 10.1002/glia.22899. 26250643 PMC4832258

[glia70074-bib-0044] Martini, R. , J. Zielasek , K. V. Toyka , K. P. Giese , and M. Schachner . 1995. “Protein Zero (P0)‐Deficient Mice Show Myelin Degeneration in Peripheral Nerves Characteristic of Inherited Human Neuropathies.” Nature Genetics 11: 281–286. 7581451 10.1038/ng1195-281

[glia70074-bib-0045] Misu, K. , T. Yoshihara , Y. Shikama , et al. 2000. “An Axonal Form of Charcot‐Marie‐Tooth Disease Showing Distinctive Features in Association With Mutations in the Peripheral Myelin Protein Zero Gene (Thr124Met or Asp75Val).” Journal of Neurology, Neurosurgery, and Psychiatry 69, no. 6: 806–811. 10.1136/jnnp.69.6.806. 11080237 PMC1737183

[glia70074-bib-0046] Moss, K. R. , T. S. Bopp , A. E. Johnson , and A. Hoke . 2021. “New Evidence for Secondary Axonal Degeneration in Demyelinating Neuropathies.” Neuroscience Letters 744: 135595. 10.1016/j.neulet.2020.135595. 33359733 PMC7852893

[glia70074-bib-0047] Nave, K. A. 2010a. “Myelination and Support of Axonal Integrity by Glia.” Nature 468, no. 7321: 244–252. 21068833 10.1038/nature09614

[glia70074-bib-0048] Nave, K. A. 2010b. “Myelination and the Trophic Support of Long Axons.” Nature Reviews. Neuroscience 11, no. 4: 275–283. 20216548 10.1038/nrn2797

[glia70074-bib-0049] Ostertag, C. , D. Klein , and R. Martini . 2022. “Presymptomatic Macrophage Targeting Has a Long‐Lasting Therapeutic Effect on Treatment Termination in a Mouse Model of Charcot‐Marie‐Tooth 1.” Experimental Neurology 357: 114195. 10.1016/j.expneurol.2022.114195. 35931123

[glia70074-bib-0050] Pisciotta, C. , and D. Pareyson . 2023. “Gene Therapy and Other Novel Treatment Approaches for Charcot‐Marie‐Tooth Disease.” Neuromuscular Disorders 33, no. 8: 627–635. 10.1016/j.nmd.2023.07.001. 37455204

[glia70074-bib-0051] Pisciotta, C. , P. Saveri , and D. Pareyson . 2021a. “Challenges in Treating Charcot‐Marie‐Tooth Disease and Related Neuropathies: Current Management and Future Perspectives.” Brain Sciences 11, no. 11: 1447. 10.3390/brainsci11111447. 34827446 PMC8615778

[glia70074-bib-0052] Pisciotta, C. , P. Saveri , and D. Pareyson . 2021b. “Updated Review of Therapeutic Strategies for Charcot‐Marie‐Tooth Disease and Related Neuropathies.” Expert Review of Neurotherapeutics 21, no. 6: 701–713. 10.1080/14737175.2021.1935242. 34033725

[glia70074-bib-0053] Prukop, T. , J. Stenzel , S. Wernick , et al. 2019. “Early Short‐Term PXT3003 Combinational Therapy Delays Disease Onset in a Transgenic Rat Model of Charcot‐Marie‐Tooth Disease 1A (CMT1A).” PLoS One 14, no. 1: e0209752. 10.1371/journal.pone.0209752. 30650121 PMC6334894

[glia70074-bib-0054] Sanmaneechai, O. , S. Feely , S. S. Scherer , et al. 2015. “Genotype‐Phenotype Characteristics and Baseline Natural History of Heritable Neuropathies Caused by Mutations in the MPZ Gene.” Brain 138, no. Pt 11: 3180–3192. 10.1093/brain/awv241. 26310628 PMC4643641

[glia70074-bib-0055] Shackleford, G. , L. N. Marziali , Y. Sasaki , et al. 2022. “A New Mouse Model of Charcot‐Marie‐Tooth 2J Neuropathy Replicates Human Axonopathy and Suggest Alteration in Axo‐Glia Communication.” PLoS Genetics 18, no. 11: e1010477. 10.1371/journal.pgen.1010477. 36350884 PMC9707796

[glia70074-bib-0056] Shy, M. E. , A. Jani , K. Krajewski , et al. 2004. “Phenotypic Clustering in MPZ Mutations.” Brain 127, no. Pt 2: 371–384. 10.1093/brain/awh048. 14711881

[glia70074-bib-0057] Song, W. M. , and M. Colonna . 2018. “The Identity and Function of Microglia in Neurodegeneration.” Nature Immunology 19, no. 10: 1048–1058. 10.1038/s41590-018-0212-1. 30250185

[glia70074-bib-0058] Wang, P. L. , A. K. Y. Yim , K. W. Kim , et al. 2020. “Peripheral Nerve Resident Macrophages Share Tissue‐Specific Programming and Features of Activated Microglia.” Nature Communications 11, no. 1: 2552. 10.1038/s41467-020-16355-w. PMC7242366 32439942

[glia70074-bib-0059] Ydens, E. , L. Amann , B. Asselbergh , et al. 2020. “Profiling Peripheral Nerve Macrophages Reveals Two Macrophage Subsets With Distinct Localization, Transcriptome and Response to Injury.” Nature Neuroscience 23: 676–689. 10.1038/s41593-020-0618-6. 32284604 PMC7611025

[glia70074-bib-0060] Yuan, X. , D. Klein , S. Kerscher , et al. 2018. “Macrophage Depletion Ameliorates Peripheral Neuropathy in Aging Mice.” Journal of Neuroscience: The Official Journal of the Society for Neuroscience 38, no. 19: 4610–4620. 10.1523/JNEUROSCI.3030-17.2018. 29712789 PMC6705935

[glia70074-bib-0061] Zhao, X. F. , L. D. Huffman , H. Hafner , et al. 2022. “The Injured Sciatic Nerve Atlas (iSNAT), Insights Into the Cellular and Molecular Basis of Neural Tissue Degeneration and Regeneration.” eLife 11: e80881. 10.7554/eLife.80881. 36515985 PMC9829412

[glia70074-bib-0062] Zielasek, J. , R. Martini , and K. V. Toyka . 1996. “Functional Abnormalities in P0‐Deficient Mice Resemble Human Hereditary Neuropathies Linked to P0 Gene Mutations.” Muscle & Nerve 19: 946–952. 8756159 10.1002/(SICI)1097-4598(199608)19:8<946::AID-MUS2>3.0.CO;2-8

