# Supplementary material for: Macrophage Targeting Protects Nerve Structure and Improves Muscle Innervation in a Mouse Model of Charcot‐Marie‐Tooth 2J
Source: Glia. 2025 Aug 4;73(12):2369–85. doi: 10.1002/glia.70074 (PMC12541899; doi:10.1002/glia.70074)
Supplement: Supplementary file 1 — Data S1: Supporting Information. [file GLIA-73-2369-s002.docx]

**Figure Legends (Supp. Info. Fig.)**

**Supp. Info. Fig. 1: Macrophages in sensory saphenous nerves of TM/+ mice do not increase in number and retain their homeostatic phenotype.**

**A.** Representative double-immunohistochemical staining against the macrophage pan-marker F4/80 (red) and the homeostatic marker CD163 (green) on cross-sections of femoral nerves from 18-month-old WT mice. White dashed circles represent the area of femoral quadriceps nerves, while grey dashed circles demarcate saphenous nerves. Nuclei are labelled with DAPI. Scale bar, 50μm.

**B.** Representative double-immunohistochemical staining against F4/80 (red) and the activation marker Gpnmb (green) on cross-sections of femoral nerves from 18-month-old WT mice. White dashed circles represent the area of femoral quadriceps nerves, while grey dashed circles demarcate saphenous nerves. Nuclei are labelled with DAPI. Scale bar, 50μm.

**C.** Analysis of F4/80-positive profiles in saphenous nerves reveals that macrophage numbers remain stable in TM/+ and WT mice across the investigated time points.

**D.** Quantification of CD163-expressing F4/80-positive macrophages. The proportion of homeostatic macrophages in WT saphenous nerves remains constant (~85%) between 12 and 18 months of age and is also not altered in TM/+ mice during disease progression.

**E.** Quantification of Gpnmb-expressing F4/80-positive macrophages. Activated macrophages are barely detectable in sensory saphenous nerves of WT and TM/+ mice across the investigated time points.

Two-way ANOVA and Tukey’s post hoc tests: *p* > 0.05.

**Supp. Info. Fig. 2: PLX5622 treatment effects on macrophages in sensory saphenous nerves**

**A.** Schematic representation of the treatment regimen using PLX5622 (CSF-1R inhibitor, 300 mg/kg). P0T124M (=TM/+) mice were treated by chow containing PLX5622 from 12 to 18 months of age (=TM/+ + PLX5622). Wild-type (WT) and TM/+ control mice received normal chow during the experiment. Mice were subsequently analyzed for functional and histopathological studies at 18M of age. Image (icons) created with BioRender.

**B.** Determination of F4/80-positive profiles in saphenous nerves shows that macrophage numbers remain unaltered in TM/+ compared to WT mice. PLX5622 treatment leads to a significant reduction in the number of macrophages in TM/+ mice.

**C.** Quantification of the percentage of CD163-expressing F4/80-positive profiles demonstrates that the proportion of homeostatic macrophages is not altered in sensory saphenous nerves of TM/+ compared to WT mice. PLX5622 treatment does not affect the percentage of CD163-expressing F4/80-macrophages in TM/+ mice.

**D.** Quantification of the percentage of Gpnmb-expressing F4/80-positive profiles demonstrates that activated macrophages are barely detectable in sensory saphenous nerves of TM/+ and WT mice. PLX5622 treatment does not affect the proportion of Gpnmb-expressing F4/80-macrophages in TM/+ mice.

**E.** Representative double-immunohistochemical staining against the macrophage pan-marker F4/80 (red) and the homeostatic marker Mgl1 (green) on a femoral nerve cross-section from an 18-month-old WT mouse. White dashed circle represents the area of femoral quadriceps nerve, while grey dashed circle demarcates the saphenous nerve. Nuclei are labelled with DAPI. Scale bar, 50μm.

**F.** Quantification of the percentage of Mgl1-expressing F4/80-positive macrophages in femoral quadriceps nerves of WT and TM/+ mice at 18 months of age. PLX5622 treatment does not alter the decreased proportion of homeostatic macrophages in femoral quadriceps nerves of TM/+ mice.

**G.** Quantification of the percentage of Mgl1-expressing F4/80-positive profiles in saphenous nerves at 18 months of age demonstrates that the proportion of homeostatic macrophages is not altered in sensory saphenous nerves of TM/+ compared to WT mice. PLX5622 treatment does not affect the percentage of Mgl1-expressing F4/80-macrophages in TM/+ mice.

One-way ANOVA and Tukey’s post hoc test (B, C, F, G), Kruskal-Wallis and Dunn's multiple comparisons test (D): ** *p* < 0.01; *** *p* < 0.001.

**Supp. Info. Fig. 3:** **Electrophysiological recordings do not show significant alterations in TM/+ mutants compared to age-matched WT mice.**

**A.** Measurement of nerve conduction velocity (NCV) demonstrates a mild, non-significant reduction in 18-month-old TM/+ control mice compared to WT littermates. PLX5622 treatment prevents the reduction of NCV in TM/+ mice.

**B.** F wave latency remains unchanged in TM/+ mice compared to WT littermates and is not affected by PLX5622 treatment.

**C., D.** Proximal (**C**) and distal (**D**) compound muscle action potentials (CMAP) amplitudes of 18-month-old mice. Both parameters remain unaltered in TM/+ mice compared to WT littermates and are not affected by PLX5622 treatment.

One-way ANOVA and Tukey’s post hoc test: *p* > 0.05.
