# Supplementary figures and images for: Macrophage Targeting Protects Nerve Structure and Improves Muscle Innervation in a Mouse Model of Charcot‐Marie‐Tooth 2J
Source: Glia. 2025 Aug 4;73(12):2369–85. doi: 10.1002/glia.70074 (PMC12541899; doi:10.1002/glia.70074)

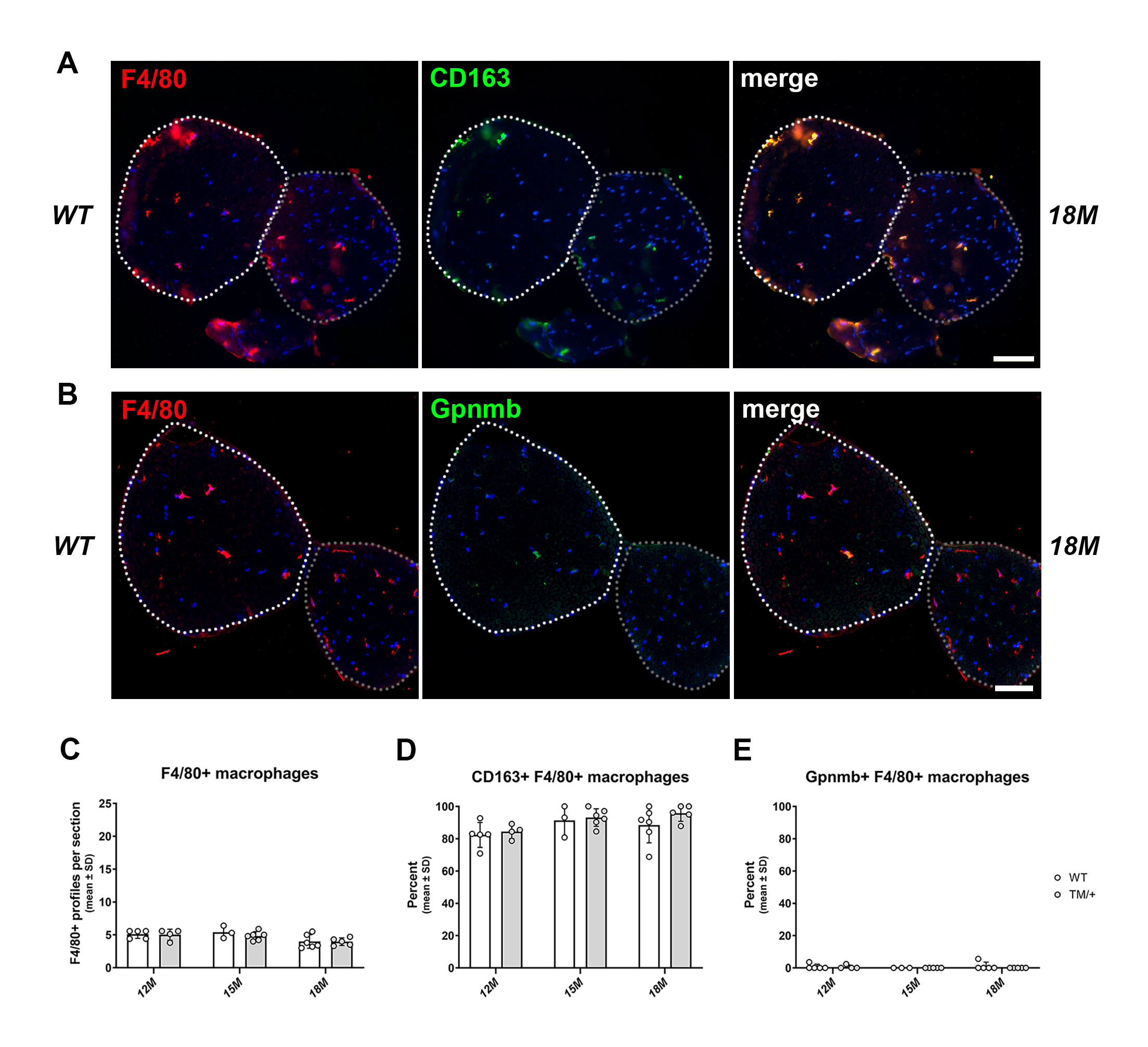

Supplement: Supplementary file 2 — Figure S1: Macrophages in sensory saphenous nerves of TM/+ mice do not increase in number and retain their homeostatic phenotype. [file GLIA-73-2369-s001.tif]

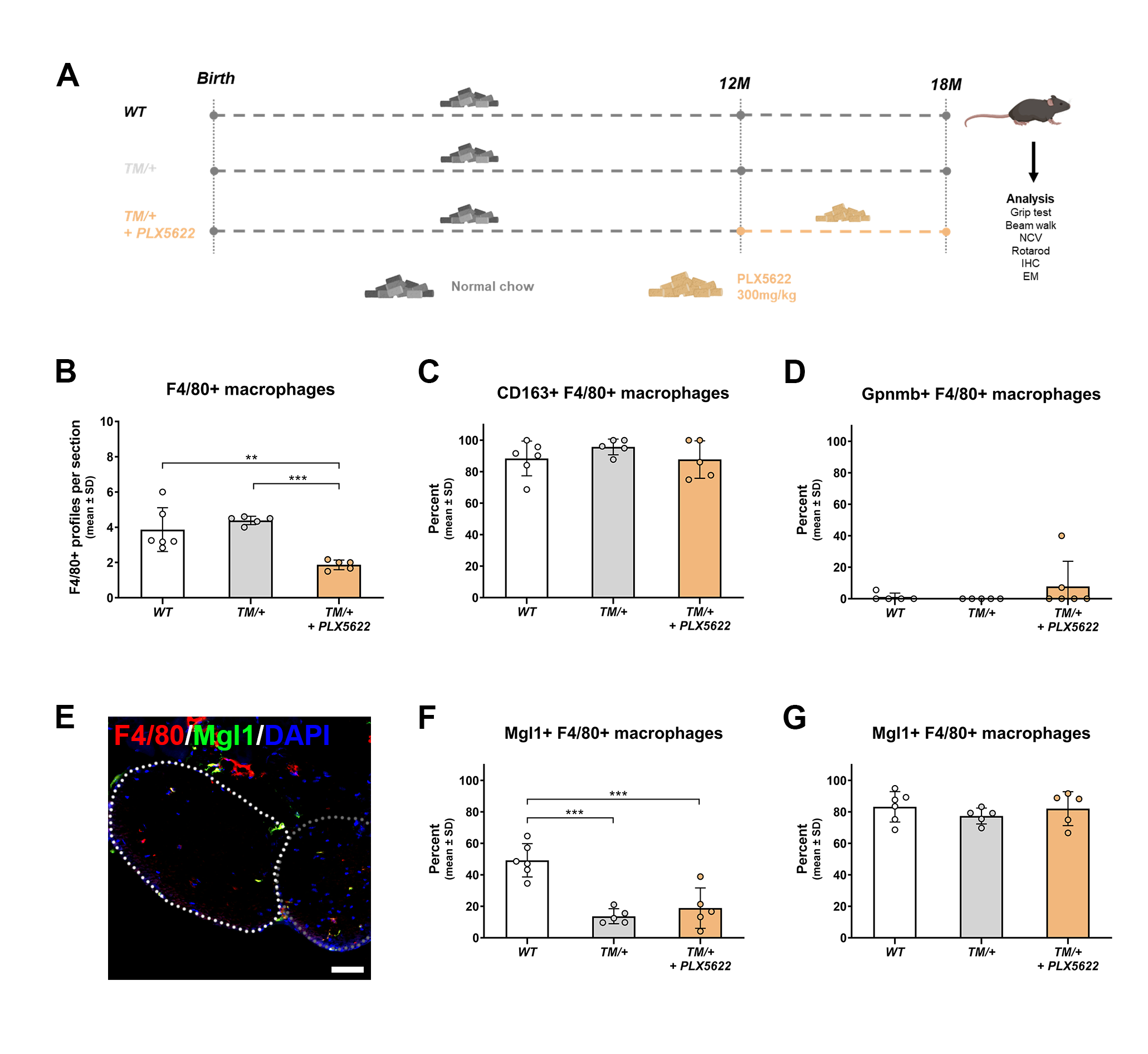

Supplement: Supplementary file 3 — Figure S2: PLX5622 treatment effects on macrophages in sensory saphenous nerves. [file GLIA-73-2369-s003.tif]

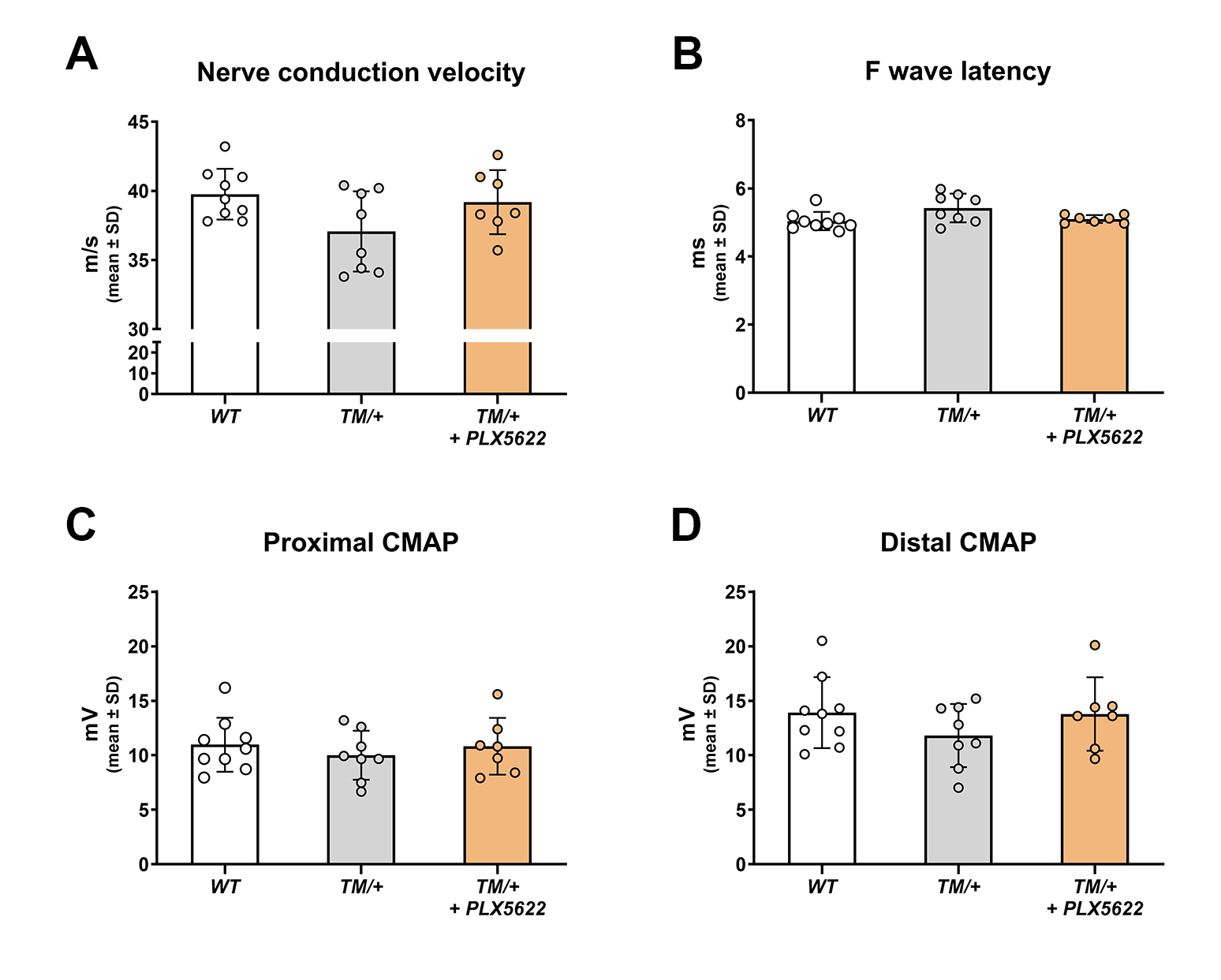

Supplement: Supplementary file 4 — Figure S3: Electrophysiological recordings do not show significant alterations in TM/+ mutants compared to age‐matched WT mice. [file GLIA-73-2369-s004.tif]
